# Modulation of the Serum Metabolome by the Short-Chain Fatty Acid Propionate: Potential Implications for Its Cholesterol-Lowering Effect

**DOI:** 10.3390/nu16142368

**Published:** 2024-07-22

**Authors:** Johann Roessler, Friederike Zimmermann, Paul Schumann, Vanasa Nageswaran, Pegah Ramezani Rad, Sven Schuchardt, David M. Leistner, Ulf Landmesser, Arash Haghikia

**Affiliations:** 1 Department of Cardiology, University Hospital St Josef-Hospital Bochum, Ruhr University Bochum, 44791 Bochum, Germany; 2 Department of Cardiology, Angiology and Intensive Care, Deutsches Herzzentrum der Charité, Campus Benjamin Franklin, 12203 Berlin, Germany; 3 DZHK (German Centre for Cardiovascular Research), Partner Site Berlin, 10785 Berlin, Germany; 4 Friede Springer Cardiovascular Prevention Center at Charité, 12203 Berlin, Germany; 5 Department of Bio and Environmental Analytics, Fraunhofer Institute for Toxicology and Experimental Medicine, 30625 Hannover, Germany; 6 Medizinische Klinik 3—Kardiologie und Angiologie, Universitätsklinikum Frankfurt am Main, 60590 Frankfurt am Main, Germany; 7 Berlin Institute of Health (BIH), 10178 Berlin, Germany

**Keywords:** gut microbiota, serum metabolome, metabolomics, cholesterol, short-chain fatty acids, bile acids, LC-MS, cardiovascular disease

## Abstract

(1) Background: Dyslipidemia represents a major risk factor for atherosclerosis-driven cardiovascular disease. Emerging evidence suggests a close relationship between cholesterol metabolism and gut microbiota. Recently, we demonstrated that the short-chain fatty acid (SCFA) propionate (PA) reduces serum cholesterol levels through an immunomodulatory mechanism. Here, we investigated the effects of oral PA supplementation on the human serum metabolome and analyzed changes in the serum metabolome in relation to the cholesterol-lowering properties of PA. (2) Methods: The serum metabolome of patients supplemented with either placebo or propionate orally for 8 weeks was assessed using a combination of flow injection analysis-tandem (FIA-MS/MS) as well as liquid chromatography (LC-MS/MS) and mass spectrometry using a targeted metabolomics kit (MxP^®^Quant 500 kit: BIOCRATES Life Sciences AG, Innsbruck, Austria). A total of 431 metabolites were employed for further investigation in this study. (3) Results: We observed a significant increase in distinct bile acids (GCDCA: fold change = 1.41, DCA: fold change = 1.39, GUDCA: fold change = 1.51) following PA supplementation over the study period, with the secondary bile acid DCA displaying a significant negative correlation with the serum cholesterol levels. (4) Conclusions: Oral supplementation with PA modulates the serum metabolome with a particular impact on the circulatory bile acid profile. Since cholesterol and bile acid metabolism are interconnected, the elevation of the secondary bile acid DCA may contribute to the cholesterol-lowering effect of PA.

## 1. Introduction

Cardiovascular disease driven by atherosclerosis remains a leading cause of death globally [1]. Well-established risk factors for atherosclerotic cardiovascular disease include distinct comorbidities such as hypertension, diabetes mellitus and dyslipidemia, as well as behavioral factors (e.g., smoking, lack of exercise). Moreover, recent research has also highlighted the gut microbiota as another key modulator of atherosclerotic cardiovascular disease with both atherogenic and atheroprotective pathways by affecting vascular inflammation and cardiometabolism [2,3,4,5,6].

The gut microbiome contains a vast number of genes, likely surpassing the human genome [3] with a wide range of metabolic functions [4,5,6]. Importantly, the gut microbiota participates in nutrient breakdown, resulting in the production of bioactive metabolites that regulate signaling pathways affecting cardiovascular health [7].

Recently, we demonstrated that the short-chain fatty acid (SCFA) propionate (PA), produced by gut microbes through bacterial fermentation of dietary fibers, critically regulates cholesterol metabolism [2]. In particular, we found that PA regulates the expression of the major cholesterol transporter NPC1L1 in the intestine by increasing intestinal IL-10 production, ultimately resulting in attenuated atherosclerosis in ApoE^−/−^-mice [2]. Furthermore, we conducted a randomized placebo-controlled clinical trial, in which oral supplementation with PA over 8 weeks resulted in a significant decrease in total cholesterol and LDL-cholesterol compared to placebo [2].

Notably, the production of bioactive metabolites by the gut microbiota extends beyond nutrient breakdown, since endogenous compounds released into the gut, such as bile acids, are metabolized by gut bacteria too [8,9]. For example, the primary bile acids cholic acid (CA) and chenodeoxycholic acid (CDCA) are released into the gut and can undergo bacterial dihydroxylation and deconjugation, resulting in the formation of secondary bile acids, predominantly lithocholic acid (LCA) and ursodeoxycholic acid (UDCA) from CDCA and deoxycholic acid (DCA) from CA [8,9]. The secondary bile acids can be re-conjugated with either glycine or taurine to form secondary conjugated bile acids, such as glyco-ursodeoxycholic acid (GUDCA) [8,9]. While some of these secondary bile acids are reabsorbed in the intestine and participate in enterohepatic circulation, they also impact endogenous metabolic pathways beyond this cycle [10], potentially influencing cardiovascular health or disease.

Here, we conducted a sub-analysis of the previous study [2] reporting for the first time on alterations in the human serum metabolome following oral supplementation with PA for 8 weeks. Moreover, we explored the relationship between changes in the serum metabolome and the cholesterol-lowering effect of PA. Our findings offer valuable insights into the regulatory role of the SCFA PA on the circulatory metabolome, providing a deeper understanding of its cholesterol-lowering and atheroprotective effects.

## 2. Materials and Methods

### 2.1. Human Studies

As previously described, we conducted a monocentric, double-blind, randomized and placebo-controlled clinical trial to assess the potential LDL-cholesterol-lowering effect of oral supplementation with PA in individuals with elevated serum cholesterol levels [2]. From July 2018 to June 2020, a total of 62 patients were continuously enrolled and randomly assigned in a 1:1 ratio to receive either placebo or PA (500 mg) orally twice a day for 8 weeks, respectively. Serum samples for metabolomic analysis were collected from each patient at the beginning (0 weeks, T1) and at the end of the study (8 weeks, T2) [2]. Significant intraindividual increase in serum PA levels upon oral supplementation was determined by stable isotope dilution gas chromatography tandem mass spectrometry (GC-MS/MS) in multiple reaction monitoring (MRM) mode, as previously reported [2]. At the timepoint of blood withdrawal, patients were instructed to be fasting, and serum samples were stored at −80 °C until further analysis.

This study was conducted in accordance with the Declaration of Helsinki, German laws and ICH: E6 (R2) guidelines, and approval by the ethics committee of Charité-Universitätsmedizin Berlin (EA4/165/16) was obtained before initiation [2]. This study was registered at https://clinicaltrials.gov/ (identifier code: NCT03590496) [2].

### 2.2. Metabolite Profiling

Analysis of the serum metabolome was performed at the Fraunhofer Institute for Toxicology and Experimental Medicine (ITEM), Hannover, Germany, operating a targeted metabolomics kit (MxP^®^Quant 500 kit: BIOCRATES Life Sciences AG, Innsbruck, Austria) according to manufacturer’s protocols, as described previously [2,11]. Briefly, after preprocessing, samples were analyzed using flow-injection analysis-tandem mass spectrometry (FIA-MS/MS) on a SCIEX 5500 QTrap^TM^ (SCIEX, Darmstadt, Germany) for lipids and liquid chromatography-tandem mass spectrometry (LC-MS/MS) for small molecules utilizing an Agilent 1290 Infinity II liquid chromatography (Santa Clara, CA, USA) linked with a SCIEX 5500 QTrap^TM^ employing multiple reaction monitoring (MRM) to detect the analytes. After the preprocessing and normalization of data, the peak-integration and calculation of metabolite concentration were conducted with METIDQ^TM^ software (Biocrates, Innsbruck, Austria). 

Amino acids, distinct amino-acid-related metabolites, distinct biogenic amines, ceramides, bile acids, sphingolipids, cholesteryl ester, diacylglycerols, distinct fatty acids, distinct indole derivatives, glycerophospholipids and triacylglycerols (in total 430 metabolites) were utilized for further investigation in this study.

### 2.3. Statistical Analysis

Statistical analysis was conducted with Prism Version 8.4.1 (GraphPad Software Inc., San Diego, CA, USA).

Kolmogorov–Smirnov and Shapiro–Wilk tests were used to determine the distribution of data, and results were expressed as mean (µmol/L) ± standard error of the mean (SEM). Paired Student’s *t*-test was used to compare means between baseline (0 weeks = T1) and end-of-study (8 weeks = T2) metabolomic data within the respective study group for normally distributed data, while the Wilcoxon test was used for data significantly deviating from a Gaussian distribution.

For correlation analysis between corresponding metabolites and lipoproteins, absolute differences (Δ) in serum concentration between the two timepoints (T2 − T1) were calculated and subjected to Pearson correlation for normally distributed data or Spearman correlation for data significantly deviating from a Gaussian distribution.

Statistical significance was assumed at two-sided *p*-value ≤ 0.05.

## 3. Results

### 3.1. Baseline Characteristics

Out of the initially 62 enrolled patients, 58 completed the study, and among them, a total of 55 patients (placebo: *n* = 28; PA: *n* = 27) with sufficient serum material were included in this sub-analysis (Supplementary Figure S1). Notably, the baseline characteristics remained well matched (Table 1). The mean age of the cohort was 50.4 years (±11.6), with a higher proportion of female participants in both groups. The mean BMI was slightly elevated with 27.1 (±4.3), with no significant difference between the study groups. Importantly, total cholesterol and LDL-cholesterol levels were comparable between the study groups, although there was a slightly significant difference in HDL-cholesterol levels, which were higher in the placebo group.

### 3.2. Impact of Oral Supplementation of PA on the Serum Metabolome in Study Participants

**Figure 1:**
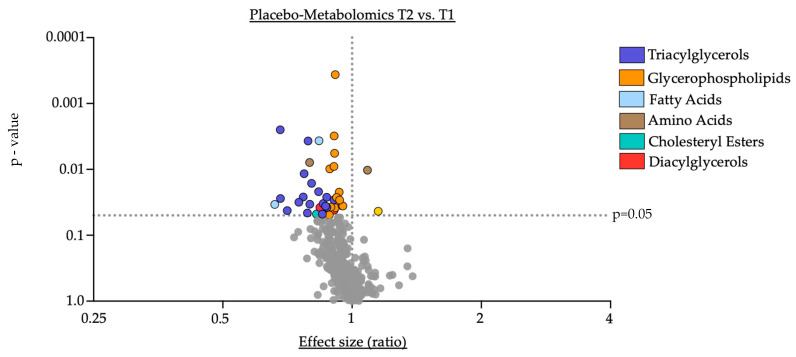
Placebo-Metabolomics T2 vs. T1. Data are presented on the *x*-axis as fold change, calculated as the ratio between serum concentration (µmol/L) at T2 divided by serum concentration at T1. Data are further outlined with the individual *p*-value in the *y*-axis, determined by comparison of means of serum concentration between the two timepoints using paired Student’s *t*-test or Wilcoxon test where eligible. Grey dots indicate not significantly altered metabolites.

**Figure 2:**
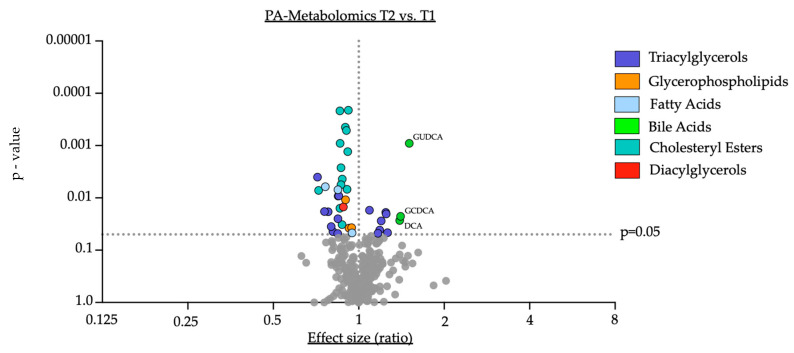
PA-Metabolomics T2 vs. T1. Data are presented in the *x*-axis as fold change, calculated as the ratio between serum concentration (µmol/L) at T2 divided by serum concentration at T1. Individual *p*-values are provided on the *y*-axis, determined by a comparison of means of serum concentration between the two timepoints using paired Student’s *t*-test or Wilcoxon test where eligible. Grey dots indicate not significantly altered metabolites. Key: GUDCA—Glycoursodeoxycholic acid; DCA—deoxycholic acid; GCDCA—Glycochenodeoxycholic acid.

Changes in the concentration of serum metabolites were analyzed over the duration of the study for each group (Figure 1 and Figure 2).

Within the placebo group, we observed a significant reduction in certain triacylglycerols (e.g., TG (48:1): fold change = 0.68, *p* = 0.003, see also Supplementary Table S1) and glycerophospholipids (e.g., PC ae C40:3: fold change = 0.91, *p* = ≤0.001, see also Supplementary Table S1). Further, the amino acid aspartate (Asp: fold change 0.79, *p* = 0.009), arachidonic acid (AA: fold change = 0.84, *p* = 0.004), 3-indolepropionic acid (3-IPA: fold change = 0.66, *p* = 0.034), two diacylglycerols (DG (16:0_16:1): fold change = 0.84, *p* = 0.038; DG (16:0_18:1): fold change = 0.91, *p* = 0.042) and the cholesteryl ester CE (20:0) (fold change = 0.83, *p* = 0.049) were significantly reduced in the serum at the end of the study compared to the corresponding baseline levels in the placebo group. Conversely, the amino acid glutamine (Gln: fold change = 1.09, *p* = 0.01) and a single ceramide (Cer (d16:1/22:0): fold change = 1.15, *p* = 0.043) displayed a significant elevated serum concentration at the end of the study compared to baseline in the placebo group. Of note, aside from the cholesteryl ester CE (20:0) the serum concentrations of the remaining investigated cholesteryl esters and bile acids remained unchanged throughout the study period (Table 2).

Within the PA group, similar to the placebo group, we observed a significant reduction in distinct triacylglycerols (e.g., TG (56:8): fold change = 0.72, *p* = 0.004, see also Supplementary Table S2), glycerophospholipids (e.g., PC aa 40:3: fold change = 0.89, *p* = 0.011, see also Supplementary Table S2), arachidonic acid (AA: fold change = 0.85, *p* = 0.007) and a single diacylglycerol (DG (18:2_18:3): fold change = 0.88, *p* = 0.015). Of note, certain triacylglycerols appeared to increase in their serum concentration (e.g., TG (18:0_36:2): fold change = 1.25, *p* = 0.02, see also Supplementary Table S2) after 8 weeks of PA supplementation compared to baseline. Furthermore, the polyunsaturated fatty acid eicopentaeonic acid (EPA: fold change = 0.76, *p* = 0.006) and the biogenic amine β-alanine (Beta-Alanine: fold change = 0.95, *p* = 0.047) were significantly reduced in their serum concentration at the end of the study compared to baseline in the PA group. Importantly and consistent with previously reported changes in lipoprotein fractions [2], the majority of measured cholesteryl esters (e.g., CE (18:2): fold change = 0.92, *p* = ≤0.001) were significantly downregulated upon PA treatment compared to baseline in the PA group (Table 3). Interestingly, upon PA supplementation, we observed a significant increase in the primary conjugated bile acid glycochenodeoxycholic acid (GCDCA) (GCDCA: fold change = 1.41, *p* = 0.023) as well as the secondary bile acid deoxycholic acid (DCA) (DCA: fold change = 1.39, *p* = 0.027) and the secondary conjugated bile acid glycoursodeoxycholic acid (GUDCA) (GUDCA: fold change = 1.51, *p* = ≤0.001) (Table 3).

### 3.3. Correlation of Shifts in Serum Bile Acids upon PA Supplementation with Its Cholesterol-Lowering Effect

Given the close interplay between bile acid and cholesterol metabolism, we further evaluated whether changes in serum concentrations of bile acids correlate with changes in lipoprotein fractions upon PA supplementation. For this, a correlation analysis (Pearson correlation and Spearman correlation, respectively, for eligible data) between the Δ of upregulated bile acids and downregulated lipoprotein fractions within the PA group was performed.

We found a significant positive correlation between the increase in the primary conjugated bile acid GCDCA with the increase in the secondary conjugated bile acid GUDCA (Spearman r: Δ-GUDCA vs. Δ-GCDCA = 0.60, *p* = ≤0.001). Both bile acids are derived from the same substrate (CDCA), while GUDCA is formed by bacterial metabolism of CDCA.

On the other hand, no correlation could be found between the increase in conjugated primary and secondary bile acid and the increase in the secondary bile acid DCA (Spearman r: Δ-DCA vs Δ-GCDCA = 0.06, *p* = 0.758; Spearman r: Δ-DCA vs. Δ-GUDCA = 0.21, *p* = 0.218), which ultimately represents a downstream product of CA by bacterial metabolism.

The conjugated primary and secondary bile acids further failed to show a correlation with the changes in the different lipoprotein fractions upon PA supplementation (Spearman r: Δ-GCDCA vs. Δ-TC = −0.14, *p* = 0.492; Spearman r: Δ-GCDCA vs Δ-LDL-C = −0.30, *p* = 0.124; Spearman r: Δ-GCDCA vs. Δ-HDL-C = 0.09, *p* = 0.649; Spearman r: Δ-GUDCA vs. Δ-TC = 0.19, *p* = 0.344; Spearman r: Δ-GUDCA vs. Δ-LDL-C = 0.04, *p* = 0.854; Spearman r: Δ-GUDCA vs. Δ-HDL-C = 0.01, *p* = 0.961).

**Figure 3:**
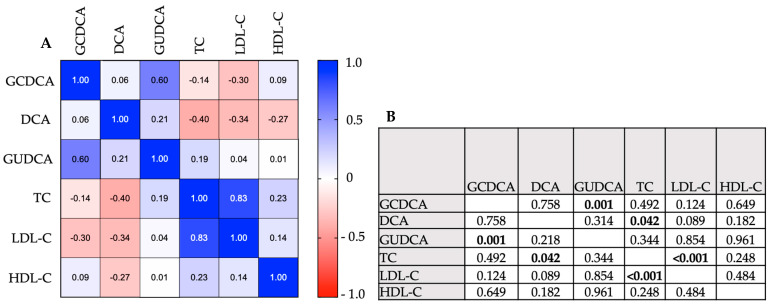
Correlation analysis between bile acids and cholesterol levels upon PA treatment. (**A**) Data are presented as Pearson/Spearman’s r in the correlation coefficient matrix. +1.0 indicates a positive correlation, −1.0 indicates a negative correlation. (**B**) Pearson/Spearman’s correlation—*p*-values. Bold values display significant *p*-values. Key: GUDCA—Glycoursodeoxycholic acid; DCA—deoxycholic acid; GCDCA—Glycochenodeoxycholic acid; TC—total cholesterol; LDL-C—low-density lipoprotein-cholesterol; HDL-C—high-density lipoprotein-cholesterol.

However, the increase in the secondary unconjugated bile acid DCA displayed a significant negative correlation with changes in total cholesterol upon PA supplementation (Pearson r: Δ-DCA vs. Δ-TCA = −0.40, *p* = 0.042) and further tended towards a negative correlation with changes in low-density lipoprotein-cholesterol (LDL-C) (Pearson r: Δ-DCA vs. Δ-LDL-C = −0.34, *p* = 0.089) upon PA supplementation (Figure 3).

## 4. Discussion

To our knowledge, this is the first study investigating the effect of the SCFA PA on the serum metabolome in humans and provides several important observations:

I. Oral supplementation with PA led to a significant downregulation of cholesteryl esters in serum, supporting prior reports of PA-induced downregulation of lipoprotein fractions by PA [2].
II. PA supplementation resulted in significant shifts in the serum metabolome with an increase in distinct bile acids (GCDCA, DCA and GUDCA), indicating a critical role for PA in modulating the circulatory bile acid profile.
III. The increase in the secondary bile acid DCA inversely correlates with the cholesterol-lowering effect of PA in humans, suggesting potential implications for the PA-related regulation of cholesterol metabolism by DCA.

These findings underscore the significant impact of oral PA supplementation on the serum metabolome, characterized by decreased levels of multiple cholesteryl esters and increased levels of distinct bile acids. This aligns with our recent findings [2] regarding the cholesterol-lowering effect of PA. The current study adds to this observation and suggests that the secondary bile acid DCA may have significant implications for the cholesterol-regulating effect of PA.

Cholesterol taken up by hepatocytes undergoes metabolism through either a classical or alternative pathway to form the primary bile acids, mainly CA or CDCA. These bile acids are conjugated with glycine or taurine to enhance their solubility and are subsequently stored in the gallbladder until they are released into the small intestine via the common bile duct in response to food intake [8].

We observed an elevation in GCDCA, a glycin-conjugated primary bile acid, following PA supplementation. Interestingly, it has been reported that, in addition to a reduced concentration of total bile acids in serum, a reduced level of GCDCA is predictive for coronary artery disease in humans [12]. Since we did not observe a correlation between cholesterol and GCDCA levels in the serum, it is plausible that GCDCA is either indirectly reduced in patients with atherosclerotic coronary artery disease or may exert its potential atheroprotective effects via cholesterol-independent pathways.

The increase in primary conjugated bile acid GCDCA following PA supplementation further suggests a potential modulation of hepatic bile acid synthesis by PA. This may involve increased bile acid synthesis via the alternative pathway, since we did not observe an upregulation of the rate limiting enzyme (cholesterol 7α-hydroxylase (Cyp7a1)) in the classical pathway in ApoE^−/−^-mice supplemented with PA in a recent work [2]. 

Inside the gut, bile acids aid in the solubilization of dietary fatty acids and lipophilic vitamins, thereby facilitating their absorption across the epithelium. Thereafter, bile acids recirculate to the liver, closing the so-called enterohepatic circulation [8], which was long believed to be their primary physiologic function. However, gut bacteria can metabolize primary bile acids, forming the secondary bile acids [8], which have been reported to mediate physiological functions beyond the enterohepatic circulation [10] and may contribute to the cholesterol-lowering effect of PA.

Interestingly, a study by Wang et al. showed impaired intestinal cholesterol absorption following oral DCA supplementation in humans, resulting in reduced plasma LDL-C [13], which supports our hypothesis that a specifically altered bile acid profile may contribute to the cholesterol-lowering effect of PA. Furthermore, the authors found increased intralumenal subphase concentration of phospholipids upon DCA supplementation, which could contribute to reduced cholesterol absorption, since phospholipids such as phosphatidylcholine inhibit intestinal cholesterol absorption [13]. The impaired intestinal cholesterol absorption due to elevated intralumenal DCA concentrations may additionally contribute to decreased intestinal cholesterol uptake, alongside the downregulation of the major cholesterol transporter NPC1L1 by PA supplementation [2].

Furthermore, bile acids can act as potent endogenous ligands for receptors involved in metabolic homeostasis, with the secondary bile acid DCA showing a high affinity for agonistic activation of key metabolic receptors as Takeda G-protein coupled receptor 5 (TGR5) and farnesoid X receptor (FXR) [8,9]. Watanabe et al. could show that bile acid supplementation in mice increased energy expenditure, resulting in reduced body weight and the prevention of high-fat-diet-induced changes in adipose mass, ultimately improving metabolic control [14]. This was mainly due to activation of the TGR5, leading to cyclic adenosine monophosphate (cAMP)-dependent activation of the thyroid hormone activating enzyme type 2 iothyronine deiodinase (D2) [14]. Importantly, the treatment of human skeletal myocytes with bile acids increased D2 activity and oxygen consumption [14], suggesting an elevated baseline metabolism with increased utilization of energy sources like circulatory lipids. Notably, TGR-5 agonism has been previously linked with improved dyslipidemia in rodents [15]. FXR agonism by bile acids or synthetic FXR agonists has also been linked with lowered plasma cholesterol levels and decreased hepatic steatosis [16,17], while FXR^−/−^-mice develop a dysregulated metabolic profile marked by dyslipidemia [18], underlining the physiological role of bile acids in metabolic homeostasis.

In the PA group, we further observed a significant increase in the secondary bile acid GUDCA, which has been recently linked to atheroprotective effects [19,20]. Interestingly, our results did not reveal a correlation between the rise in GUDCA and changes in cholesterol levels, despite rodent studies reporting a significant decrease in plasma cholesterol upon GUDCA supplementation [19,20]. It is important to note that the serum levels of GUDCA achieved through PA supplementation may not reach concentrations obtained with specific supplementation, which could explain the lack of correlation with cholesterol levels in our setting. Additionally, GUDCA has been shown to have beneficial cardiometabolic effects beyond cholesterol reduction, such as inhibiting foam cell formation [20] and acting as a bioactive ligand [19]. These effects are partially dependent on its antagonistic actions on intestinal FXR, unlike DCA, which acts as an FXR agonist [9,19]. Nevertheless, both secondary bile acids serve as agonists for TGR5 [9], which may play a more crucial role in the bile-acid-mediated regulation of cholesterol homeostasis. Recently, Farr et al. demonstrated a significant reduction in postprandial lipemia, including cholesterol and triglycerides, following DCA administration in mice [21]. The authors concluded that the lipid lowering effect of DCA is primarily mediated by its TGR-5 agonism, as evidenced by its persistence in FXR^−/−^-mice, while another analyzed bile acid (tauro-cholic acid) failed to improve postprandial lipemia in these mice [21].

The upregulation of the secondary bile acids DCA and GUDCA may be attributed to changes in microbial metabolism of primary bile acids upon PA supplementation. It is conceivable that the consumption of SCFAs could modify the gut microenvironment, promoting the activity of microbes involved in the metabolism of primary bile acids. This modulation could occur through mechanisms such as pH-regulation or immunomodulation, as demonstrated in previous studies [22,23]. Moreover, the modulation of the microenvironment by PA could enhance bile-acid—receptor interactions, e.g., due to conformational changes impacting receptor-ligand kinetics. On the other hand, the potential inhibition of enzymatic processes due to PA supplementation could also contribute to the observed changes in the serum metabolome. For example, a cell-culture-based experiment reported that SCFA supplementation inhibited the activity of the lecithin-cholesterol acyltransferase, an enzyme that mediates the esterification of cholesterol, thereby enhancing its solubility for blood transport [24]. However, it should be noted that propionate was not directly studied in this experiment [24]. 

Furthermore, it has been shown that among other secondary bile acids, TGR-5 activation by DCA exhibits anti-inflammatory properties both in vitro and in vivo. This effect is characterized by a reduction in the production of pro-inflammatory cytokines in macrophages, leading to ameliorated inflammation in a mouse model of inflammatory bowel disease [25]. Moreover, the activation of bile acid receptors (TGR-5, FXR) on immune cells promotes an anti-inflammatory phenotype, marked by an increase in M2 macrophages and Treg cells in the gut mucosa, along with the upregulation of anti-inflammatory cytokines such as IL-10 [23]. Importantly, as we previously demonstrated, the cholesterol-lowering effect of PA is at least partly mediated by the increased production of the anti-inflammatory cytokine IL-10, which ultimately reduces NPC1L1 expression [2].

Of note, we observed significant changes in the serum level of distinct metabolites, particularly triacylglycerols and glycerophospholipids, in both the treatment and control groups over the study period. Since these changes occurred regardless of the treatment, we concluded they are nonspecific and may be attributed to factors such as the participants nutrient intake and the timing of blood collection. Triglycerides are mainly transported in chylomicrons and very low-density lipoproteins, which are formed from absorbed intestinal triglycerides, and the serum concentration varies throughout the day based on meal timings [26]. Additionally, the synthesis and degradation of both lipid classes follow a circadian rhythm [27,28].

Furthermore, the partially reported high standard errors indicate the wide variability of the respective data, which should be considered when interpreting the results. Increased data variability due to high standard errors can reduce the power of the statistical analysis may obscuring potential significant effects.

It should be noted that the results reported here display a correlation between changes in serum metabolites and the cholesterol-lowering effect of PA treatment, which does not necessarily imply a direct or indirect causal relationship. Based on the presented data, it cannot be directly concluded whether the altered bile acid profile is a cause or a consequence of the cholesterol-lowering effect of PA. It has previously been reported that the use of the cholesterol-lowering drug atorvastatin also mediates a shift in serum bile acid profile in mice, although no correlation of bile acids and the cholesterol-lowering effect of atorvastatin was provided [29]. Of note, a study conducted in rat hepatocytes has reported that treatment with propionate specifically enhances bile acid synthesis and secretion [30]. Thus, the potential implications of an altered bile acid profile by PA on its cholesterol-lowering effects via the aforementioned pathways are conceivable.

However, further research is necessary to examine the precise mechanisms underlying the potential cholesterol-lowering effects of bile acids, in particular, of the secondary bile acid DCA. Additionally, further research is necessary to investigate the mechanisms by which PA influences the human serum metabolome.

## 5. Conclusions

Oral supplementation with the gut-microbiota-derived SCFA PA not only reduces serum cholesterol levels [2] but also significantly impacts the serum metabolome. Our findings suggest a regulatory role for PA in the circulatory bile acid profile, particularly with regard to the secondary bile acid DCA, which shows a significant inverse correlation with the cholesterol-lowering effect of PA. Since cholesterol and bile acid metabolism are interconnected, the modulation of the circulatory bile acid profile by PA with a specific increase in the secondary bile acid DCA may contribute to its cholesterol-lowering effect.

## Figures and Tables

**Table 1:** Study patients’ demographics.

	All Patients (*n* = 55)	Placebo (*n* = 28)	PA (*n* = 27)	*p*-Value
Age, y	50.4 (±11.6)	51.8 (±11.1)	49.1 (±11.9)	0.395
Females	39 (70.1%)	21 (75%)	18 (66.7%)	0.778
Body Mass Index *	27.1 (±4.3)	26.4 (±3.7)	27.7 (±4.7)	0.268
Medical history				
Diabetes	1	0	1	0.313
Hypertension	11	4	7	0.378
Cholesterol levels (mg/dL)				
Total (mg/dL)	256.9 (±43.3)	263.1 (±36.1)	250.4 (±48.7)	0.284
LDL (mg/dL)	184.7 (±41)	188.3 (±34.9)	181.2 (±46.3)	0.53
HDL (mg/dL)	67.2 (±20.8)	72.8 (±20.1)	61.3 (±19.8)	0.047

Data are presented as mean ± standard error of mean (SEM). * Body mass index was calculated by weight in kilograms divided by the square of height in meters.

**Table 2:** Metabolomic data of cholesteryl esters and bile acids of the Placebo group (T1 vs. T2).

Metabolite	Placebo_T1	Placebo_T2	*p*-Value
Cholesteryl Esters			
CE(14:0)	35.9 (±11.5)	35.7 (±9.5)	0.899
CE(14:1)	0.9 (±0.8)	0.9 (±0.8)	0.866
CE(15:0)	14.2 (±5.8)	14.1 (±4.9)	0.39
CE(15:1)	0.8 (±0.3)	0.7 (±0.2)	0.054
CE(16:0)	282.5 (±62.3)	275.5 (±50.4)	0.587
CE(16:1)	93.6 (±43.9)	89.9 (±41.2)	0.476
CE(17:0)	10.6 (±3.3)	10.5 (±3.1)	0.567
CE(17:1)	8.2 (±4.2)	7.9 (±4.3)	0.55
CE(18:0)	22.8 (±5.6)	22.5 (±6.8)	0.789
CE(18:1)	576.6 (±160.1)	565 (±180.7)	0.641
CE(18:2)	1630 (±341.5)	1592.5 (±313)	0.471
CE(18:3)	100.2 (±44.1)	90.6 (±34.7)	0.124
CE(20:0)	2 (±0.6)	1.6 (±0.5)	0.048
CE(20:1)	0.8 (±0.2)	0.7 (±0.2)	0.175
CE(20:3)	38.5 (±12.8)	39.8 (±13.7)	0.497
CE(20:4)	336.3 (±111.9)	347.8 (±130.8)	0.504
CE(20:5)	126.3 (±66)	127.9 (±66.3)	0.65
CE(22:2)	0.2 (±0)	0.2 (±0.1)	0.117
CE(22:5)	3.4 (±0.9)	3.4 (±1)	0.959
CE(22:6)	53.3 (±22.6)	53.1 (±19)	0.526
Bile Acids			
Cholic acid	0.1 (±0.1)	0.4 (±0.4)	0.999
Deoxycholic acid	0.3 (±0.3)	0.3 (±0.3)	0.772
Glycocholic acid	0.2 (±0.2)	0.2 (±0.4)	0.419
Glycochenodeoxycholic acid	0.4 (±0.3)	0.5 (±0.6)	0.399
Glycodeoxycholic acid	0.2 (±0.3)	0.3 (±0.3)	0.417
Glycolithocholic acid	0.01 (±0)	0.01 (±0)	0.999
Glycolithocholic acid sulfate	0.2 (±0.1)	0.2 (±0.2)	0.915
Glycoursodeoxycholic acid	0.1 (±0.1)	0.1 (±0.1)	0.241
Taurocholic acid	0.1 (±0.1)	0.1 (±0.2)	0.296
Taurochenodeoxycholic acid	0.1 (±0.1)	0.1 (±0.1)	0.827
Taurodeoxycholic acid	0.1 (±0.1)	0.1 (±0.1)	0.768

Data are presented as mean (µmol/L) ± standard error of mean (SEM).

**Table 3:** Metabolomic data of cholesteryl ester and bile acids of the PA group (T1 vs. T2).

Metabolite	Placebo_T1	Placebo_T2	*p*-Value
Cholesteryl Esters			
CE(14:0)	35.4 (±12.9)	30.4 (±12.7)	0.016
CE(14:1)	1.1 (±0.9)	0.9 (±0.8)	0.192
CE(15:0)	14.8 (±5.4)	12.5 (±4.8)	0.009
CE(15:1)	0.8 (±0.2)	0.7 (±0.3)	0.134
CE(16:0)	270.5 (±51.4)	242.8 (±50.7)	<0.001
CE(16:1)	95.9 (±42.3)	82.5 (±38.1)	0.001
CE(17:0)	10.5 (±3.9)	9.2 (±3)	0.004
CE(17:1)	8 (±3.5)	7 (±3.1)	0.032
CE(18:0)	22.1 (±6.3)	20.2 (±6.8)	0.001
CE(18:1)	540.3 (±150)	491.7 (±149.8)	0.007
CE(18:2)	1597 (±322.4)	1468.9 (±313.1)	<0.001
CE(18:3)	96 (±36.2)	82.3 (±36.9)	<0.001
CE(20:0)	2 (±0.6)	1.5 (±0.5)	0.007
CE(20:1)	0.8 (±0.2)	0.7 (±0.3)	0.211
CE(20:3)	37.8 (±15.6)	32.7 (±12.8)	0.006
CE(20:4)	318.3 (±112.7)	288.1 (±105)	0.001
CE(20:5)	131.1 (±64.4)	99 (±51.1)	<0.001
CE(22:2)	0.2 (±0.1)	0.1 (±0)	0.184
CE(22:5)	3.1 (±1)	2.9 (±1)	0.08
CE(22:6)	51.5 (±20.8)	44.6 (±15.9)	0.003
Bile Acids			
Cholic acid	0.3 (±0.3)	0.3 (±0.2)	0.61
Deoxycholic acid	0.2 (±0.2)	0.3 (±0.2)	0.027
Glycocholic acid	0.1 (±0.2)	0.2 (±0.1)	0.059
Glycochenodeoxycholic acid	0.3 (±0.3)	0.4 (±0.3)	0.023
Glycodeoxycholic acid	0.1 (±0.2)	0.2 (±0.2)	0.111
Glycolithocholic acid	0.01 (±0)	0.01 (±0)	0.707
Glycolithocholic acid sulfate	0.2 (±0.2)	0.2 (±0.2)	0.312
Glycoursodeoxycholic acid	0.01 (+0)	0.1 (±0)	<0.001
Taurocholic acid	0.01 (±0)	0.01 (±0)	0.242
Taurochenodeoxycholic acid	0.01 (±0)	0.1 (±0.1)	0.158
Taurodeoxycholic acid	0.01 (±0)	0.01 (±0.1)	0.469

Data are presented as mean (µmol/L) ± standard error of mean (SEM).

## Data Availability

Data are contained within the article and Supplementary Materials. The data presented in this study are available in Supplementary Tables S1 and S2. Further data present in this study are available on request from the corresponding author.

## Supplementary Materials

The following supporting information can be downloaded at: https://www.mdpi.com/article/10.3390/nu16142368/s1, Supplementary Information S1 (Table S1—placebo metabolomics T1 vs. T2, Table S2—PA metabolomics T1 vs. T2; Figure S1—study enrollment), supplementary Information S2 (blank, quality control data, representative chromatograms of bile acids).
