# Supplementary material for: Modulation of the Serum Metabolome by the Short-Chain Fatty Acid Propionate: Potential Implications for Its Cholesterol-Lowering Effect
Source: Nutrients. 2024 Jul 22;16(14):2368. doi: 10.3390/nu16142368 (PMC11280296; doi:10.3390/nu16142368)
Supplement: Supplementary file 1 [file nutrients-16-02368-s001.zip › supplementary information 1.pdf]

Supplementary Material

Supplementary Table S1. Metabolomic data and analysis of the Placebo-group (T1 vs. T2).

Metabolite	Placebo_T1	Placebo_T2	*p*-Value
**Amino Acids**			
Alanine	350 (± 89.3)	337.8 (± 86.6)	0.423
Arginine	60.6 (± 20.8)	64.5 (± 16.8)	0.247
Asparagine	63.3 (± 29.7)	67 (± 25)	0.22
Aspartic acid	5.3 (± 1.4)	4.2 (± 0.6)	0.008
Cysteine	41.4 (± 4.2)	50.4 (± 10.2)	<0.001
Glutamic acid	638 (± 106.1)	693.3 (± 127.6)	0.01
Glutamine	79 (± 26.4)	45.7 (± 21.9)	<0.001
Glycine	216.3 (± 60.5)	223 (± 67)	0.473
Histidine	78.4 (± 9.4)	77.5 (± 10)	0.677
Isoleucine	62 (± 14.7)	62.3 (± 16.9)	0.924
Leucine	112.7 (± 21.9)	112 (± 25.4)	0.868
Lysine	160.6 (± 32.2)	156.3 (± 31.5)	0.499
Methionine	17.9 (± 7.1)	20.3 (± 7.7)	0.37
Phenylalanine	54.4 (± 9)	55.9 (± 8.5)	0.482
Proline	160 (± 52.6)	161.6 (± 48.5)	0.866
Serine	91.5 (± 21.6)	94.9 (± 18.2)	0.423
Threonine	107 (± 31.1)	112.1 (± 34.7)	0.486
Tryptophan	59.2 (± 11.4)	55.2 (± 12.6)	0.067
Tyrosine	55.7 (± 14.8)	54.8 (± 13.3)	0.749
Valine	197.1 (± 37.4)	198.3 (± 42.1)	0.89
**Ceramides**			
Cer(d16:1/18:0)	0.1 (± 0)	0.1 (± 0)	0.866
Cer(d16:1/20:0)	0.1 (± 0.1)	0.1 (± 0)	0.069
Cer(d16:1/22:0)	0.4 (± 0.2)	0.5 (± 0.1)	0.043
Cer(d16:1/23:0)	0.2 (± 0.1)	0.2 (± 0.1)	0.247
Cer(d16:1/24:0)	0.4 (± 0.1)	0.4 (± 0.1)	0.716
Cer(d18:1/14:0)	0.1 (± 0)	0.1 (± 0)	0.336
Cer(d18:1/16:0)	0.6 (± 0.1)	0.6 (± 0.1)	0.971
Cer(d18:1/18:0)	0.2 (± 0.1)	0.2 (± 0.1)	0.451
Cer(d18:1/20:0)	0.2 (± 0)	0.2 (± 0)	0.415
Cer(d18:1/22:0)	1.0 (± 0.3)	1.0 (± 0.3)	0.826
Cer(d18:1/23:0)	1.2 (± 0.4)	1.2 (± 0.5)	0.724
Cer(d18:1/24:0)	3.1 (± 0.9)	3.1 (± 0.9)	0.698
Cer(d18:1/24:1)	1.8 (± 0.5)	1.8 (± 0.6)	0.854
Cer(d18:1/25:0)	1.0 (± 0.6)	1.0 (± 0.7)	0.483
Cer(d18:1/26:0)	0.1 (± 0)	0.1 (± 0)	0.335
Cer(d18:1/26:1)	0.01 (± 0)	0.01 (± 0)	0.596
Cer(d18:2/16:0)	0.1 (± 0.1)	0.1 (± 0)	0.719
Cer(d18:2/18:0)	0.1 (± 0)	0.1 (± 0)	0.969
Cer(d18:2/20:0)	0.1 (± 0)	0.1 (± 0)	0.335
Cer(d18:2/22:0)	0.3 (± 0.1)	0.3 (± 0.1)	0.972
Cer(d18:2/23:0)	0.2 (± 0.1)	0.2 (± 0.1)	0.157
Cer(d18:2/24:0)	0.7 (± 0.2)	0.7 (± 0.2)	0.731
Cer(d18:2/24:1)	0.4 (± 0.1)	0.4 (± 0.2)	0.416
**Bile Acids**			
Cholic acid	0.1 (± 0.1)	0.4 (± 0.4)	0.999
Deoxycholic acid	0.3 (± 0.3)	0.3 (± 0.3)	0.772

Glycocholic acid	0.2 (± 0.2)	0.2 (± 0.4)	0.419
Glycochenodeoxycholic acid	0.4 (± 0.3)	0.5 (± 0.6)	0.399
Glycodeoxycholic acid	0.2 (± 0.3)	0.3 (± 0.3)	0.417
Glycolithocholic acid	0.01 (± 0)	0.01 (± 0)	0.999
Glycolithocholic acid sulfate	0.2 (± 0.1)	0.2 (± 0.2)	0.915
Glycoursodeoxycholic acid	0.1 (± 0.1)	0.1 (± 0.1)	0.241
Taurocholic acid	0.1 (± 0.1)	0.1 (± 0.2)	0.296
Taurochenodeoxycholic acid	0.1 (± 0.1)	0.1 (± 0.1)	0.827
Taurodeoxycholic acid	0.1 (± 0.1)	0.1 (± 0.1)	0.768
**Sphingolipids**			
SM (OH) C14:1	7.8 (± 2)	7.6 (± 1.8)	0.296
SM (OH) C16:1	3.7 (± 1)	3.6 (± 1)	0.901
SM (OH) C22:1	12.3 (± 2.2)	12.2 (± 2.1)	0.696
SM (OH) C22:2	10.9 (± 2.5)	10.6 (± 2.3)	0.314
SM (OH) C24:1	1 (± 0.2)	1.1 (± 0.3)	0.582
SM C16:0	110.9 (± 16.8)	111.8 (± 17.8)	0.804
SM C16:1	16.9 (± 3.5)	16.7 (± 3.8)	0.62
SM C18:0	23.7 (± 5.9)	23.5 (± 5.7)	0.511
SM C18:1	10.7 (± 2.4)	10.5 (± 2.6)	0.907
SM C20:2	0.3 (± 0.1)	0.3 (± 0.1)	0.734
SM C24:0	17.2 (± 3.3)	16.9 (± 2.7)	0.562
SM C24:1	42.9 (± 10)	41.5 (± 9.4)	0.376
SM C26:0	0.1 (± 0)	0.1 (± 0)	0.756
SM C26:1	0.3 (± 0.1)	0.3 (± 0.1)	0.476
**Cholesteryl Esters**			
CE(14:0)	35.9 (± 11.5)	35.7 (± 9.5)	0.899
CE(14:1)	0.9 (± 0.8)	0.9 (± 0.8)	0.866
CE(15:0)	14.2 (± 5.8)	14.1 (± 4.9)	0.39
CE(15:1)	0.8 (± 0.3)	0.7 (± 0.2)	0.054
CE(16:0)	282.5 (± 62.3)	275.5 (± 50.4)	0.587
CE(16:1)	93.6 (± 43.9)	89.9 (± 41.2)	0.476
CE(17:0)	10.6 (± 3.3)	10.5 (± 3.1)	0.567
CE(17:1)	8.2 (± 4.2)	7.9 (± 4.3)	0.55
CE(18:0)	22.8 (± 5.6)	22.5 (± 6.8)	0.789
CE(18:1)	576.6 (± 160.1)	565 (± 180.7)	0.641
CE(18:2)	1630 (± 341.5)	1592.5 (± 313)	0.471
CE(18:3)	100.2 (± 44.1)	90.6 (± 34.7)	0.124
CE(20:0)	2 (± 0.6)	1.6 (± 0.5)	0.048
CE(20:1)	0.8 (± 0.2)	0.7 (± 0.2)	0.175
CE(20:3)	38.5 (± 12.8)	39.8 (± 13.7)	0.497
CE(20:4)	336.3 (± 111.9)	347.8 (± 130.8)	0.504
CE(20:5)	126.3 (± 66)	127.9 (± 66.3)	0.65
CE(22:2)	0.2 (± 0)	0.2 (± 0.1)	0.117
CE(22:5)	3.4 (± 0.9)	3.4 (± 1)	0.959
CE(22:6)	53.3 (± 22.6)	53.1 (± 19)	0.526
**Diacylglycerols**			
DG(16:0_16:1)	0.8 (± 0.4)	0.7 (± 0.3)	0.038
DG(16:0_18:1)	3.8 (± 1.4)	3.5 (± 2.2)	0.042
DG(16:0_18:2)	1.3 (± 0.5)	1.4 (± 0.6)	0.932
DG(16:1_18:2)	0.6 (± 0.2)	0.6 (± 0.3)	0.743
DG(17:0_18:1)	0.6 (± 0.2)	0.6 (± 0.3)	0.804
DG(18:1_18:1)	3.8 (± 1.4)	3.9 (± 2.1)	0.88

DG(18:1_18:2)	6.5 (± 2.5)	6.6 (± 2.8)	0.98
DG(18:1_18:3)	0.7 (± 0.4)	0.6 (± 0.3)	0.702
DG (18:1_20:0)	0.5 (± 0.3)	0.5 (± 0.3)	0.738
DG(18:1_20:1)	0.1 (± 0.1)	0.1 (± 0.1)	0.987
DG(18:1_20:3)	0.3 (± 0.2)	0.3 (± 0.2)	0.735
DG(18:1_20:4)	0.3 (± 0.2)	0.3 (± 0.1)	0.611
DG(18:2_18:2)	2.8 (± 1.8)	2.8 (± 1.2)	0.827
DG(18:2_18:3)	0.4 (± 0.2)	0.3 (± 0.1)	0.195
DG(18:2_20:0)	0.1 (± 0)	0.1 (± 0)	0.571
**Glycerophospholipids**			
LysoPC a C16:0	102 (± 20.7)	92.9 (± 20.9)	0.006
LysoPC a C16:1	3.1 (± 1.1)	2.8 (± 1.3)	0.065
LysoPC a C17:0	2 (± 0.5)	1.8 (± 0.4)	0.038
LysoPC a C18:0	32 (± 7)	28.4 (± 5.8)	0.01
lysoPC a C18:1	24.4 (± 7.7)	22.8 (± 12.8)	0.022
lysoPC a C18:2	33.5 (± 12.6)	32.7 (± 18.6)	0.297
lysoPC a C20:3	2.4 (± 0.8)	2.3 (± 1.2)	0.184
lysoPC a C20:4	6.4 (± 2.4)	6.1 (± 2.2)	0.086
lysoPC a C28:1	0.6 (± 0.3)	0.6 (± 0.2)	0.122
PC aa C28:1	5 (± 1.2)	4.9 (± 1.2)	0.594
PC aa C30:0	5.7 (± 1.7)	5.6 (± 1.6)	0.858
PC aa C30:2	0.1 (± 0.1)	0.1 (± 0.2)	0.573
PC aa C32:0	16.7 (± 3.6)	16.1 (± 3.4)	0.392
PC aa C32:1	21.7 (± 11.1)	19.2 (± 8.9)	0.042
PC aa C32:2	5.5 (± 1.7)	5.5 (± 1.8)	0.966
PC aa C32:3	0.7 (± 0.2)	0.7 (± 0.1)	0.282
PC aa C34:1	226.1 (± 44.5)	214.3 (± 47.9)	0.036
PC aa C34:2	363.3 (± 33)	357.2 (± 42.5)	0.37
PC aa C34:3	20.5 (± 5.6)	18.3 (± 5.7)	0.037
PC aa C34:4	2.6 (± 1)	2.4 (± 0.9)	0.206
PC aa C36:0	1.8 (± 1)	1.5 (± 0.9)	0.053
PC aa C36:1	63.2 (± 17.3)	57.4 (± 15.8)	0.029
PC aa C36:2	250 (± 34.6)	239.1 (± 42.7)	0.132
PC aa C36:3	140.5 (± 20.2)	134.4 (± 27.8)	0.147
PC aa C36:4	190.6 (± 45.8)	183.6 (± 41.2)	0.066
PC aa C36:5	36.5 (± 19.8)	33.6 (± 15.1)	0.716
PC aa C36:6	1.2 (± 0.5)	1.1 (± 0.4)	0.371
PC aa C38:0	2.7 (± 0.9)	2.6 (± 0.7)	0.312
PC aa C38:1	0.6 (± 0.4)	0.5 (± 0.3)	0.201
PC aa C38:3	53.1 (± 13.7)	50 (± 13.5)	0.084
PC aa C38:4	106.4 (± 25.5)	101.3 (± 26.2)	0.096
PC aa C38:5	59.7 (± 14)	56.3 (± 14.6)	0.184
PC aa C38:6	88.1 (± 31.6)	84.8 (± 24.9)	0.626
PC aa C40:2	0.3 (± 0.1)	0.2 (± 0.1)	0.11
PC aa C40:3	0.5 (± 0.1)	0.5 (± 0.1)	0.582
PC aa C40:4	3 (± 0.7)	2.8 (± 0.7)	0.027
PC aa C40:5	8.7 (± 1.8)	7.9 (± 1.9)	0.009
PC aa C40:6	26.5 (± 8.8)	24.5 (± 7.8)	0.056
PC aa C42:0	0.5 (± 0.1)	0.5 (± 0.1)	0.367
PC aa C42:1	0.2 (± 0.1)	0.2 (± 0.1)	0.558
PC aa C42:2	0.2 (± 0.1)	0.2 (± 0)	0.072
PC aa C42:4	0.2 (± 0)	0.1 (± 0)	0.103

PC aa C42:5	0.3 (± 0.1)	0.3 (± 0.1)	0.166
PC aa C42:6	0.5 (± 0.1)	0.4 (± 0.1)	0.415
PC ae C30:0	0.5 (± 0.1)	0.5 (± 0.1)	0.939
PC ae C30:1	0.3 (± 0.1)	0.3 (± 0.1)	0.896
PC ae C30:2	0.1 (± 0.1)	0.1 (± 0.1)	0.12
PC ae C32:1	3.2 (± 0.8)	3.1 (± 0.7)	0.712
PC ae C32:2	0.8 (± 0.2)	0.8 (± 0.2)	0.621
PC ae C34:0	1.8 (± 0.5)	1.8 (± 0.5)	0.963
PC ae C34:1	11.9 (± 2.5)	11.6 (± 2.7)	0.271
PC ae C34:2	13.8 (± 3.2)	13.1 (± 3.1)	0.185
PC ae C34:3	9 (± 2.3)	8.4 (± 2)	0.146
PC ae C36:0	0.9 (± 0.2)	0.9 (± 0.2)	0.241
PC ae C36:1	9.4 (± 2.2)	8.9 (± 2.1)	0.152
PC ae C36:2	16.3 (± 4.1)	16 (± 4.1)	0.617
PC ae C36:3	7.9 (± 1.7)	7.5 (± 1.7)	0.126
PC ae C36:4	17.8 (± 5.2)	16.8 (± 3.8)	0.128
PC ae C36:5	11.8 (± 3.5)	11.1 (± 2.7)	0.194
PC ae C38:0	2.5 (± 0.7)	2.3 (± 0.6)	0.263
PC ae C38:1	0.5 (± 0.3)	0.4 (± 0.3)	0.204
PC ae C38:2	1.9 (± 0.5)	1.7 (± 0.5)	0.049
PC ae C38:3	4.2 (± 1)	3.9 (± 1)	0.066
PC ae C38:4	13.3 (± 3)	13.1 (± 3)	0.693
PC ae C38:5	18.4 (± 3.8)	17.6 (± 3.3)	0.145
PC ae C38:6	7.9 (± 2.5)	7.6 (± 2)	0.421
PC ae C40:1	1.4 (± 0.3)	1.3 (± 0.2)	0.128
PC ae C40:2	2.2 (± 0.5)	2.1 (± 0.5)	0.126
PC ae C40:3	1.1 (± 0.2)	1 (± 0.2)	<0.001
PC ae C40:4	2.1 (± 0.4)	2 (± 0.4)	0.036
PC ae C40:5	3.2 (± 0.6)	3.1 (± 0.7)	0.16
PC ae C40:6	4.6 (± 1.3)	4.4 (± 1.1)	0.339
PC ae C42:1	0.3 (± 0.1)	0.3 (± 0)	0.029
PC ae C42:2	0.6 (± 0.1)	0.5 (± 0.1)	0.003
PC ae C42:3	0.7 (± 0.1)	0.7 (± 0.1)	0.134
PC ae C42:4	0.7 (± 0.1)	0.7 (± 0.2)	0.585
PC ae C42:5	1.7 (± 0.3)	1.7 (± 0.4)	0.287
PC ae C44:3	0.1 (± 0)	0.1 (± 0)	0.101
PC ae C44:4	0.3 (± 0.1)	0.3 (± 0.1)	0.721
PC ae C44:5	1.4 (± 0.3)	1.3 (± 0.3)	0.304
PC ae C44:6	1 (± 0.2)	0.9 (± 0.2)	0.196
**Triacylglycerols**			
TG(46:2)	3.1 (± 1.8)	2.8 (± 2.9)	0.085
TG(48:0)	4.7 (± 3.8)	3.9 (± 2.9)	0.087
TG(48:1)	32.1 (± 22.4)	26.8 (± 18.1)	0.095
TG(48:2)	17.2 (± 8.7)	15.5 (± 9.7)	0.306
TG(48:3)	3.5 (± 1.7)	3.3 (± 2.5)	0.591
TG(49:1)	0.8 (± 0.6)	0.7 (± 0.5)	0.08
TG(49:2)	1 (± 0.6)	0.8 (± 0.6)	0.264
TG(50:1)	6.2 (± 3.3)	5.6 (± 3.5)	0.297
TG(50:2)	31.6 (± 13.8)	29.9 (± 18)	0.573
TG(50:3)	21.7 (± 10.5)	20.7 (± 13.1)	0.576
TG(50:4)	6.8 (± 4.6)	6.6 (± 5.5)	0.706
TG(52:4)	0.8 (± 0.3)	0.7 (± 0.5)	0.192

TG(52:5)	0.7 (± 0.3)	0.7 (± 0.4)	0.162
TG(44:1)	3.1 (± 3)	2.3 (± 2.2)	0.089
TG(44:2)	0.9 (± 0.7)	0.7 (± 0.5)	0.108
TG(46:2)	3.6 (± 3)	3.4 (± 5.4)	0.132
TG(48:0)	35.5 (± 49.4)	24.2 (± 28.7)	0.028
TG(48:1)	60.6 (± 53.5)	46.7 (± 36)	0.026
TG(48:2)	24 (± 16)	19.2 (± 12.5)	0.08
TG(48:3)	3.6 (± 2.3)	3 (± 2.3)	0.022
TG(49:1)	8 (± 5.9)	6.8 (± 4.2)	0.126
TG(49:2)	2.9 (± 1.7)	2.5 (± 1.2)	0.129
TG(50:0)	24.1 (± 22.3)	20 (± 18.6)	0.101
TG(50:1)	170.5 (± 118.6)	146.9 (± 110.9)	0.06
TG(50:2)	122.6 (± 66)	107.2 (± 56.4)	0.038
TG(50:3)	32.5 (± 14.7)	28.6 (± 13.6)	0.079
TG(50:4)	3.9 (± 2.1)	3.3 (± 2)	0.117
TG(51:1)	5 (± 3.4)	4.3 (± 2.7)	0.15
TG(51:2)	6.5 (± 3.8)	5.8 (± 3)	0.22
TG(51:3)	2.1 (± 0.9)	2 (± 0.8)	0.455
TG(52:2)	287.8 (± 114)	273.5 (± 135)	0.54
TG(52:3)	215.5 (± 77.5)	207.6 (± 80.3)	0.595
TG(52:4)	63.2 (± 28.9)	60.5 (± 28)	0.532
TG(52:5)	9.5 (± 4.5)	8.9 (± 5.2)	0.458
TG(52:6)	1.3 (± 0.6)	1.2 (± 0.8)	0.647
TG(53:3)	0.5 (± 0.2)	0.5 (± 0.2)	0.209
TG(54:1)	1.5 (± 1)	1.4 (± 1)	0.453
TG(54:2)	3.3 (± 1.5)	3.4 (± 2.3)	0.556
TG(54:3)	4.4 (± 1.7)	4.3 (± 2.1)	0.702
TG(54:4)	6.2 (± 2.3)	5.8 (± 2.7)	0.388
TG(54:5)	6.7 (± 2.7)	6.3 (± 2.8)	0.297
TG(54:6)	3.8 (± 1.7)	3.6 (± 1.7)	0.59
TG(54:7)	1.3 (± 0.6)	1.3 (± 0.7)	0.961
TG(56:6)	4.4 (± 1.6)	4.2 (± 1.7)	0.487
TG(56:7)	4.3 (± 2)	4.2 (± 1.7)	0.774
TG(56:8)	1.5 (± 0.8)	1.5 (± 0.8)	0.921
TG(44:1)	1.2 (± 1)	1 (± 1)	0.177
TG(46:2)	3.5 (± 2.8)	2.5 (± 2.3)	0.042
TG(48:1)	12.3 (± 16.3)	8.4 (± 9.5)	0.003
TG(48:2)	17 (± 13.5)	13.2 (± 9.4)	0.012
TG(48:3)	4.3 (± 2.2)	3.7 (± 2.4)	0.135
TG(49:2)	2.4 (± 1.4)	1.9 (± 1.3)	0.016
TG(50:1)	8.9 (± 7)	7 (± 5.7)	0.004
TG(50:2)	66.9 (± 39.9)	57.3 (± 37.6)	0.033
TG(50:3)	30.9 (± 14.3)	27.6 (± 14.8)	0.135
TG(50:4)	6.2 (± 2.9)	5.7 (± 3.1)	0.334
TG(52:2)	7.6 (± 3.6)	7.2 (± 5.3)	0.574
TG(52:3)	36.6 (± 13.3)	35.2 (± 20.5)	0.726
TG(52:4)	25.8 (± 10.5)	24.4 (± 11.1)	0.55
TG(52:5)	7.8 (± 4.3)	7.2 (± 3.3)	0.441
TG(52:6)	1.7 (± 1)	1.5 (± 0.9)	0.337
TG(54:4)	0.7 (± 0.3)	0.6 (± 0.3)	0.636
TG(54:5)	1 (± 0.4)	1 (± 0.5)	0.715
TG(54:6)	1.5 (± 0.6)	1.4 (± 0.6)	0.342

TG(49:1)	1.6 (± 1.2)	1.2 (± 0.8)	0.031
TG(51:1)	4.5 (± 2.8)	4 (± 2.4)	0.268
TG(51:2)	2.9 (± 1.5)	2.7 (± 1.2)	0.338
TG(51:3)	0.7 (± 0.3)	0.6 (± 0.3)	0.152
TG(53:3)	3.8 (± 1.7)	3.7 (± 1.4)	0.657
TG(53:4)	1.4 (± 0.6)	1.3 (± 0.4)	0.109
TG(49:2)	1.9 (± 1.2)	1.6 (± 0.9)	0.062
TG(51:2)	5.7 (± 1.4)	5.1 (± 2.8)	0.244
TG(51:3)	2.8 (± 1.4)	2.5 (± 1.2)	0.294
TG(51:4)	0.6 (± 0.3)	0.6 (± 0.2)	0.877
TG(53:4	2.1 (± 0.9)	2.1 (± 0.9)	0.969
TG(53:5)	0.7 (± 0.3)	0.7 (± 0.3)	0.608
TG(53:6)	0.4 (± 0.2)	0.3 (± 0.2)	0.222
TG(55:6)	0.1 (± 0.1)	0.1 (± 0.1)	0.499
TG(55:7)	0.1 (± 0.1)	0.1 (± 0.1)	0.39
TG(51:4)	0.3 (± 0.1)	0.3 (± 0.1)	0.598
TG(51:5)	0.3 (± 0.1)	0.3 (± 0.1)	0.209
TG(53:4)	0.4 (± 0.2)	0.4 (± 0.2)	0.555
TG(53:5)	0.3 (± 0.1)	0.3 (± 0.1)	0.406
TG(53:6)	0.5 (± 0.2)	0.5 (± 0.3)	0.959
TG(55:7)	0.2 (± 0.1)	0.2 (± 0.1)	0.22
TG(55:8)	0.2 (± 0.1)	0.2 (± 0.1)	0.835
TG(55:9)	0.1 (± 0.1)	0.1 (± 0)	0.478
TG(48:0)	2.8 (± 2.8)	2.2 (± 1.7)	0.046
TG(48:1)	1.8 (± 1.5)	1.6 (± 1.6)	0.091
TG(50:0)	6.5 (± 8)	5.1 (± 6.4)	0.225
TG(50:1)	7.7 (± 7.5)	6.2 (± 6.1)	0.034
TG(50:2)	2 (± 1.3)	1.8 (± 1.2)	0.294
TG(52:2)	12.7 (± 7.7)	12.1 (± 9.6)	0.399
TG(52:3)	2.8 (± 1.4)	2.6 (± 2)	0.062
TG(54:1)	4.6 (± 3.1)	4.9 (± 5.3)	0.741
TG(54:2)	20.2 (± 11.2)	21.9 (± 22.1)	0.679
TG(54:3)	17.4 (± 7.7)	18.3 (± 11.1)	0.438
TG(54:4)	6 (± 2.9)	6.2 (± 3.4)	0.915
TG(54:5)	1.1 (± 0.5)	1.1 (± 0.7)	0.362
TG(54:6)	1.2 (± 0.5)	1.2 (± 0.4)	0.758
TG(54:7)	0.4 (± 0.2)	0.4 (± 0.2)	0.814
TG(44:1)	2.8 (± 2.3)	3 (± 5.7)	0.854
TG(44:2)	3.5 (± 2.6)	3.1 (± 3.5)	0.063
TG(48:1)	40.8 (± 26.4)	35.6 (± 25.5)	0.026
TG(48:2)	25.9 (± 16)	24.2 (± 24.1)	0.074
TG(48:3)	5 (± 4.4)	5.6 (± 8.8)	0.661
TG(49:1)	7.4 (± 5.2)	6.3 (± 3.6)	0.064
TG(50:2)	106.5 (± 68.5)	92.3 (± 65.2)	0.082
TG(50:2)	133.1 (± 61.4)	118.9 (± 67.9)	0.156
TG(50:3)	30.7 (± 12.2)	28.9 (± 16.6)	0.462
TG(50:4)	3.8 (± 1.6)	3.5 (± 2.4)	0.504
TG(51:1)	5.9 (± 3.8)	5.3 (± 3)	0.282
TG(51:2)	16.9 (± 8.7)	15.7 (± 7.9)	0.453
TG(51:3)	4.9 (± 2.2)	4.7 (± 2.2)	0.608
TG(51:4)	1 (± 0.5)	0.9 (± 0.5)	0.538
TG(52:2)	479 (± 182.6)	451 (± 223.1)	0.478

TG(52:3)	244.3 (± 83.2)	233.8 (± 96.5)	0.566
TG(52:4)	43.6 (± 17.2)	41 (± 19.3)	0.508
TG(52:5)	4.9 (± 2)	4.4 (± 2.4)	0.308
TG(53:3)	7.2 (± 3.2)	7.1 (± 3.7)	0.833
TG(53:4)	2.2 (± 0.9)	2.1 (± 0.9)	0.604
TG(54:1)	5.6 (± 2.8)	5.6 (± 4.7)	0.114
TG(54:2)	47.2 (± 23)	50.9 (± 45.4)	0.695
TG(54:3)	149.6 (± 59.4)	154.2 (± 113.7)	0.469
TG(54:4)	105.4 (± 46.7)	106.8 (± 65.3)	0.339
TG(54:5)	36.7 (± 19.6)	35.2 (± 20.2)	0.169
TG(54:6)	8.6 (± 4.9)	8 (± 4.8)	0.543
TG(54:7)	1.4 (± 0.7)	1.4 (± 0.8)	0.786
TG(56:6)	7.8 (± 2.4)	7.7 (± 2.7)	0.598
TG(56:7)	4.8 (± 2)	4.8 (± 1.9)	0.94
TG(56:8)	1.2 (± 0.5)	1.2 (± 0.6)	0.978
TG(46:2)	3.5 (± 3.1)	4.1 (± 7.2)	0.64
TG(48:2)	13.5 (± 6.8)	12.5 (± 9.5)	0.053
TG(48:3)	8.3 (± 5.5)	8.3 (± 10.7)	0.81
TG(50:2)	37.5 (± 17.7)	34.5 (± 16.3)	0.192
TG(50:3)	43.8 (± 17.1)	40.2 (± 19.4)	0.271
TG(50:4)	11.2 (± 7.7)	10.4 (± 7.3)	0.393
TG(51:2)	2.1 (± 1)	1.9 (± 0.8)	0.228
TG(51:3)	5.6 (± 2.6)	5.3 (± 2.3)	0.465
TG(51:4)	1.9 (± 0.9)	1.8 (± 0.9)	0.589
TG(52:2)	25.2 (± 9.9)	23.7 (± 11)	0.374
TG(52:3)	195.7 (± 70.4)	186.1 (± 71.1)	0.455
TG(52:4)	94.2 (± 46.4)	91.2 (± 41.4)	0.679
TG(52:5)	15.7 (± 9.6)	14.5 (± 7.4)	0.459
TG(52:6)	1.9 (± 1.3)	1.6 (± 0.9)	0.314
TG(53:3)	4.1 (± 1.8)	3.9 (± 1.5)	0.464
TG(53:4)	2.4 (± 1.2)	2.3 (± 1)	0.591
TG(53:5)	0.8 (± 0.4)	0.7 (± 0.3)	0.705
TG(54:2)	2.5 (± 1.1)	2.6 (± 1.6)	0.784
TG(54:3)	21.3 (± 9.3)	22.4 (± 13.6)	0.469
TG(54:4)	54.2 (± 25.4)	54.8 (± 31.6)	0.425
TG(54:5)	34.6 (± 23.2)	33.8 (± 19.2)	0.396
TG(54:6)	14.1 (± 12.8)	14 (± 12.6)	0.362
TG(54:7)	3.5 (± 3.6)	3.7 (± 4.7)	0.292
TG(56:6)	2.3 (± 1)	2.2 (± 0.8)	0.758
TG(56:7)	3.5 (± 1.6)	3.3 (± 1.2)	0.427
TG(56:8)	1.9 (± 1)	2 (± 1)	0.654
TG(48:2)	2.3 (± 1.5)	1.9 (± 2)	0.048
TG(48:3)	5.3 (± 2.8)	4.5 (± 2.8)	0.174
TG(50:4)	7 (± 3.6)	5.9 (± 4.1)	0.188
TG(51:5)	0.3 (± 0.2)	0.2 (± 0.2)	0.532
TG(52:3)	3.5 (± 1.5)	3.1 (± 1.9)	0.089
TG(52:4)	27.8 (± 6.8)	25 (± 12.9)	0.312
TG(52:5)	14.2 (± 6.8)	12.7 (± 6.9)	0.259
TG(52:6)	2.4 (± 1.5)	2.1 (± 1.4)	0.297
TG(53:5)	0.5 (± 0.3)	0.5 (± 0.3)	0.462
TG(54:4)	3.3 (± 1.4)	3.1 (± 2.4)	0.054
TG(54:5)	11.5 (± 5.5)	10.7 (± 6.9)	0.115

TG(54:6)	7.5 (± 5.5)	6.7 (± 4.3)	0.216
TG(54:7)	2.8 (± 2.7)	2.6 (± 2.9)	0.149
TG(54:8)	0.6 (± 0.3)	0.5 (± 0.3)	0.175
TG(54:9)	0.4 (± 0.2)	0.4 (± 0.2)	0.185
TG(52:3)	0.6 (± 0.2)	0.6 (± 0.2)	0.746
TG(52:4)	0.5 (± 0.2)	0.5 (± 0.2)	0.383
TG(54:1)	1.5 (± 1.1)	1.2 (± 0.7)	0.111
TG(50:2)	0.3 (± 0.1)	0.3 (± 0.1)	0.422
TG(52:2)	1 (± 0.5)	1 (± 0.7)	0.97
TG(52:3)	0.5 (± 0.2)	0.5 (± 0.2)	0.796
TG(52:4)	0.2 (± 0.1)	0.2 (± 0.1)	0.296
TG(54:1)	0.6 (± 0.2)	0.6 (± 0.5)	0.58
TG(54:2)	3.2 (± 1.5)	3.3 (± 2.6)	0.65
TG(54:3)	1.7 (± 0.7)	1.7 (± 1)	0.957
TG(54:4)	0.4 (± 0.1)	0.4 (± 0.2)	0.266
TG(52:2)	1 (± 0.6)	0.9 (± 0.6)	0.101
TG(52:3)	1.2 (± 0.6)	1.1 (± 0.6)	0.414
TG(54:3)	3.2 (± 1.4)	3.1 (± 1.8)	0.319
TG(54:4)	1.9 (± 0.7)	1.8 (± 0.8)	0.251
TG(54:5)	0.4 (± 0.2)	0.4 (± 0.2)	0.37
TG(56:7)	0.1 (± 0)	0.1 (± 0)	0.546
TG(52:3)	1.7 (± 1)	1.6 (± 1.1)	0.376
TG(52:4)	2.1 (± 1.1)	1.9 (± 1.3)	0.373
TG(52:5)	0.6 (± 0.3)	0.6 (± 0.3)	0.973
TG(54:3)	0.9 (± 0.4)	0.9 (± 0.5)	0.257
TG(54:4)	7 (± 2.9)	6.6 (± 3.7)	0.544
TG(54:5)	3.8 (± 1.5)	3.7 (± 1.8)	0.508
TG(54:6)	0.8 (± 0.3)	0.8 (± 0.5)	0.775
TG(56:6)	1.9 (± 0.8)	2 (± 1)	0.779
TG(56:7)	0.8 (± 0.4)	0.8 (± 0.3)	0.709
TG(56:8)	0.4 (± 0.2)	0.3 (± 0.2)	0.231
TG(50:4)	1.5 (± 1.1)	1.3 (± 1.4)	0.074
TG(52:4)	3.5 (± 2.4)	2.9 (± 1.9)	0.06
TG(52:5)	3.7 (± 2.2)	3.2 (± 2.1)	0.036
TG(52:6)	1.2 (± 0.6)	1.1 (± 0.9)	0.09
TG(53:6)	0.2 (± 0.1)	0.2 (± 0.1)	0.977
TG(54:4)	2.2 (± 1.2)	2 (± 1.1)	0.124
TG(54:5)	15.4 (± 6.7)	14.6 (± 7.2)	0.139
TG(54:6)	8.4 (± 3.6)	8.1 (± 4.2)	0.319
TG(54:7)	1.5 (± 0.7)	1.4 (± 0.7)	0.442
TG(55:7)	0.1 (± 0)	0.1 (± 0)	0.517
TG(56:6)	9.1 (± 3.2)	9 (± 3.6)	0.866
TG(56:7)	5.3 (± 2)	5.2 (± 2.1)	0.192
TG(56:8)	2.1 (± 1)	2 (± 1)	0.416
TG(56:9)	0.8 (± 0.5)	0.6 (± 0.3)	0.188
TG(54:5)	0.9 (± 0.7)	0.8 (± 0.4)	0.598
TG(54:6)	5.2 (± 3.6)	4.7 (± 2.5)	0.955
TG(54:7)	2.9 (± 1.8)	2.8 (± 1.8)	0.815
TG(56:7)	2.8 (± 1.7)	2.8 (± 1.6)	0.937
TG(56:8)	1.9 (± 1)	1.9 (± 1.2)	0.995
TG(54:6)	0.01 (± 0)	0.01 (± 0)	0.075
TG(54:6)	0.1 (± 0.1)	0.1 (± 0.1)	0.794

TG(54:4)	0.4 (± 0.3)	0.4 (± 0.2)	0.191
TG(54:6)	0.1 (± 0.1)	0.1 (± 0.1)	0.651
TG(56:6)	1 (± 0.4)	1 (± 0.5)	0.805
TG(54:5)	1.3 (± 0.7)	1.2 (± 0.6)	0.319
TG(54:6)	1.4 (± 0.7)	1.3 (± 0.6)	0.376
TG(56:6)	6.6 (± 2.5)	6.5 (± 2.3)	0.796
TG(56:7)	3.6 (± 1.5)	3.6 (± 1.3)	0.901
TG(56:8)	0.7 (± 0.3)	0.7 (± 0.3)	0.791
TG(54:6)	2.7 (± 2)	2.5 (± 1.3)	0.543
TG(54:7)	3.1 (± 2.2)	3 (± 1.9)	0.832
TG(56:7)	11.9 (± 7.1)	12.5 (± 7.1)	0.683
TG(56:8)	6.3 (± 3.7)	6.9 (± 4.4)	0.552
TG(56:9)	1.2 (± 0.7)	1.4 (± 0.9)	0.418
**Various Metabolites**			
Beta-Alanine	2.4 (± 0.9)	2.3 (± 0.7)	0.339
AABA	15.3 (± 4.4)	15.4 (± 4.8)	0.459
HArg	2.4 (± 1)	2.2 (± 0.9)	0.111
HCys	6 (± 1.3)	6 (± 1.4)	0.344
SDMA	0.7 (± 0.2)	0.6 (± 0.2)	0.335
AA	4.1 (± 1.2)	3.4 (± 0.7)	0.004
DHA	6.3 (± 3.4)	5.6 (± 2.6)	0.158
EPA	0.9 (± 0.6)	0.8 (± 0.3)	0.19
3-IAA	1.9 (± 0.9)	1.7 (± 0.7)	0.227
3-IPA	1.5 (± 1.6)	1 (± 0.8)	0.034
Ind-SO4	5 (± 2.5)	5.1 (± 2.8)	0.357
H1	4570.1 (± 559)	4624.5 (± 600.4)	0.332
TMAO	6 (± 8.6)	4.9 (± 4.3)	0.409

Data are presented as mean (µmol/l) ± standard error of mean (SEM).

Supplementary Table S2. Metabolomic data and analysis of the PA-group (T1 vs. T2).

Metabolite	PA_T1	PA_T2	*p*-Value
**Amino Acids**			
Alanine	355.6 (± 105)	360 (± 70.8)	0.821
Arginine	74.8 (± 20.9)	75 (± 21.5)	0.963
Asparagine	63.3 (± 18.6)	63.5 (± 18.7)	0.64
Aspartic acid	5.7 (± 2.3)	4.9 (± 1)	0.583
Cysteine	43.6 (± 8.3)	54.9 (± 9.5)	<0.001
Glutamic acid	652 (± 86.1)	677.5 (± 100.9)	0.143
Glutamine	82.5 (± 30.9)	57.5 (± 22.4)	<0.001
Glycine	227.6 (± 75.5)	228.9 (± 65.4)	0.864
Histidine	80.7 (± 8.8)	80.7 (± 8.7)	0.999
Isoleucine	66.6 (±16)	69.6 (± 24.1)	0.398
Leucine	120.8 (27.2)	122.4 (± 36.8)	0.754
Lysine	175.3 (± 29.5)	173 (± 41)	0.718
Methionine	19.7 (± 6.8)	21.5 (± 8.7)	0.352
Phenylalanine	56.2 (± 7.8)	56.6 (± 8.4)	0.802
Proline	192.5 (± 80.5)	183.6 (± 64)	0.44
Serine	98.5 (± 16.6)	95.5 (± 13)	0.293
Threonine	108.5 (± 28.5)	109.5 (± 23)	0.865
Tryptophan	60.2 (± 12.5)	61.7 (± 13.8)	0.557
Tyrosine	64.2 (± 15)	65.6 (± (14.9)	0.679
Valine	211.3 (± 53.1)	210.8 (± 53.7)	0.959

**Ceramides**			
Cer(d16:1/18:0)	0.1 (± 0)	0.1 (± 0)	0.825
Cer(d16:1/20:0)	0.1 (± 0)	0.1 (± 0)	0.647
Cer(d16:1/22:0)	0.4 (± 0.1)	0.4 (± 0.1)	0.805
Cer(d16:1/23:0)	0.2 (± 0.1)	0.2 (± 0.1)	0.912
Cer(d16:1/24:0)	0.3 (± 0.1)	0.3 (± 0.1)	0.977
Cer(d18:1/14:0)	0.1 (± 0)	0.1 (± 0)	0.17
Cer(d18:1/16:0)	0.5 (± 0.1)	0.5 (± 0.1)	0.434
Cer(d18:1/18:0)	0.2 (± 0)	0.2 (± 0.1)	0.867
Cer(d18:1/20:0)	0.2 (± 0)	0.2 (± 0.1)	0.53
Cer(d18:1/22:0)	1 (±0.3)	1.0 (± 0.3)	0.233
Cer(d18:1/23:0)	1.2 (± 0.3)	1.2 (± 0.3)	0.746
Cer(d18:1/24:0)	3.0 (± 0.8)	3.1 (± 0.8)	0.348
Cer(d18:1/24:1)	1.8 (± 0.4)	1.8 (± 0.5)	0.772
Cer(d18:1/25:0)	1 (± 0.7)	1 (± 0.7)	0.35
Cer(d18:1/26:0)	0.1 (± 0)	0.1 (± 0)	0.833
Cer(d18:1/26:1)	0.01 (± 0)	0.01 (± 0)	0.269
Cer(d18:2/16:0)	0.1 (± 0)	0.1 (± 0)	0.671
Cer(d18:2/18:0)	0.1 (± 0)	0.1 (± 0)	0.191
Cer(d18:2/20:0)	0.1 (± 0)	0.1 (± 0)	0.424
Cer(d18:2/22:0)	0.4 (± 0.1)	0.4 (± 0.1)	0.621
Cer(d18:2/23:0)	0.2 (± 0.1)	0.2 (± 0.1)	0.602
Cer(d18:2/24:0)	0.7 (± 0.2)	0.7 (± 0.2)	0.878
Cer(d18:2/24:1)	0.4 (± 0.1)	0.4 (± 0.1)	0.773
**Bile Acids**			
Cholic acid	0.3 (± 0.3)	0.3 (± 0.2)	0.61
Deoxycholic acid	0.2 (± 0.2)	0.3 (± 0.2)	0.027
Glycocholic acid	0.1 (± 0.2)	0.2 (± 0.1)	0.059
Glycochenodeoxycholic acid	0.3 (± 0.3)	0.4 (± 0.3)	0.023
Glycodeoxycholic acid	0.1 (± 0.2)	0.2 (± 0.2)	0.111
Glycolithocholic acid	0.01 (± 0)	0.01 (± 0)	0.707
Glycolithocholic acid sulfate	0.2 (± 0.2)	0.2 (± 0.2)	0.312
Glycoursodeoxycholic acid	0.01 (+ 0)	0.1 (± 0)	<0.001
Taurocholic acid	0.01 (± 0)	0.01 (± 0)	0.242
Taurochenodeoxycholic acid	0.01 (± 0)	0.1 (± 0.1)	0.158
Taurodeoxycholic acid	0.01 (± 0)	0.01 (± 0.1)	0.469
**Sphingolipids**			
SM (OH) C14:1	7.5 (± 1.8)	7.4 (± 1.9)	0.42
SM (OH) C16:1	3.6 (± 0.8)	3.4 (± 0.9)	0.149
SM (OH) C22:1	11.8 (± 2.8)	11.4 (± 2.8)	0.281
SM (OH) C22:2	10.3 (± 2.4)	10 (± 2.6)	0.286
SM (OH) C24:1	1 (± 0.2)	1 (± 0.2)	0.087
SM C16:0	109.1 (± 20.2)	106.5 (± 19.8)	0.32
SM C16:1	16.2 (± 3.2)	15.9 (± 3.7)	0.533
SM C18:0	22.4 (± 4)	21.8 (± 4.8)	0.275
SM C18:1	10.3 (± 2.4)	10 (± 2.4)	0.355
SM C20:2	0.3 (± 0.1)	0.3 (± 0.1)	0.662
SM C24:0	16.4 (± 3.8)	16.5 (± 4.1)	0.849
SM C24:1	39.9 (± 9.1)	39.1 (± 10.6)	0.374
SM C26:0	0.1 (± 0)	0.1 (+ 0)	0.282
SM C26:1	0.3 (± 0.1)	0.3 (± 0.1)	0.956
**Cholesteryl Esters**			

CE(14:0)	35.4 (± 12.9)	30.4 (± 12.7)	0.016
CE(14:1)	1.1 (± 0.9)	0.9 (± 0.8)	0.192
CE(15:0)	14.8 (± 5.4)	12.5 (± 4.8)	0.009
CE(15:1)	0.8 (± 0.2)	0.7 (± 0.3)	0.134
CE(16:0)	270.5 (± 51.4)	242.8 (± 50.7)	<0.001
CE(16:1)	95.9 (± 42.3)	82.5 (± 38.1)	0.001
CE(17:0)	10.5 (± 3.9)	9.2 (± 3)	0.004
CE(17:1)	8 (± 3.5)	7 (± 3.1)	0.032
CE(18:0)	22.1 (± 6.3)	20.2 (± 6.8)	0.001
CE(18:1)	540.3 (± 150)	491.7 (± 149.8)	0.007
CE(18:2)	1597 (± 322.4)	1468.9 (± 313.1)	<0.001
CE(18:3)	96 (± 36.2)	82.3 (± 36.9)	<0.001
CE(20:0)	2 (± 0.6)	1.5 (± 0.5)	0.007
CE(20:1)	0.8 (± 0.2)	0.7 (± 0.3)	0.211
CE(20:3)	37.8 (± 15.6)	32.7 (± 12.8)	0.006
CE(20:4)	318.3 (± 112.7)	288.1 (± 105)	0.001
CE(20:5)	131.1 (± 64.4)	99 (± 51.1)	<0.001
CE(22:2)	0.2 (± 0.1)	0.1 (± 0)	0.184
CE(22:5)	3.1 (± 1)	2.9 (± 1)	0.08
CE(22:6)	51.5 (± 20.8)	44.6 (± 15.9)	0.003
**Diacylglycerols**			
DG(16:0_16:1)	0.9 (± 0.5)	0.9 (± 0.4)	0.755
DG(16:0_18:1)	3.8 (± 1.9)	4 (± 2.2)	0.144
DG(16:0_18:2)	1.4 (± 0.7)	1.5 (± 0.8)	0.504
DG(16:1_18:2)	0.7 (± 0.3)	0.7 (± 0.3)	0.61
DG(17:0_18:1)	0.6 (± 0.2)	0.7 (± 0.2)	0.59
DG(18:1_18:1)	4.2 (± 1.9)	4.6 (± 2.4)	0.15
DG(18:1_18:2)	7.4 (± 3.7)	7.6 (± 4.5)	0.736
DG(18:1_18:3)	0.8 (± 0.4)	0.7 (± 0.4)	0.431
DG(18:1_20:0)	0.4 (± 0.2)	0.5 (± 0.2)	0.534
DG(18:1_20:1)	0.1 (± 0.1)	0.1 (± 0.1))	0.538
DG(18:1_20:3)	0.4 (± 0.3)	0.4 (± 0.3)	0.733
DG(18:1_20:4)	0.3 (± 0.2)	0.3 (± 0.2)	0.6
DG(18:2_18:2)	3.1 (± 2)	3 (± 2.1)	0.587
DG(18:2_18:3)	0.4 (± 0.1)	0.4 (± 0.1)	0.015
DG(18:2_20:0)	0.1 (± 0)	0.1 (± 0)	0.901
**Glycerophospholipids**			
LysoPC a C16:0	91.9 (± 16)	92.5 (± 16.9)	0.844
LysoPC a C16:1	2.8 (± 0.9)	2.8 (± 1)	0.755
LysoPC a C17:0	1.8 (± 0.5)	1.8 (± 0.4)	0.27
LysoPC a C18:0	28.9 (± 7.2)	28.4 (± 6.7)	0.625
lysoPC a C18:1	20.6 (± 5)	20.7 (± 5.4)	0.855
lysoPC a C18:2	26.9 (± 7.7)	27.1 (± 7.1)	0.896
lysoPC a C20:3	1.9 (± 0.5)	2.1 (± 0.6)	0.073
lysoPC a C20:4	5.4 (± 1.6)	5.5 (± 1.9)	0.747
lysoPC a C28:1	0.5 (± 0.2)	0.6 (± 0.2)	0.406
PC aa C28:1	4.7 (± 1.3)	4.6 (± 1.4)	0.383
PC aa C30:0	4.8 (± 1.6)	5.3 (± 2.3)	0.396
PC aa C30:2	0.1 (± 0.1)	0.1 (± 0.2)	0.387
PC aa C32:0	15.2 (± 2.9)	15.4 (± 3.3)	0.778
PC aa C32:1	20.6 (± 11.7)	22.1 (± 12.4)	0.202
PC aa C32:2	5.2 (± 2.2)	5.2 (± 2.4)	0.377

PC aa C32:3	0.7 (± 0.2)	0.7 (± 0.2)	0.682
PC aa C34:1	211.3 (± 42.3)	222.3 (± 45.9)	0.074
PC aa C34:2	357 (± 52.7)	358.2 (± 62)	0.87
PC aa C34:3	19.1 (± 6.4)	18.7 (± 6.1)	0.637
PC aa C34:4	2.2 (± 0.8)	2.3 (± 1)	0.827
PC aa C36:0	1.5 (± 0.6)	1.5 (± 0.7)	0.905
PC aa C36:1	55.5 (± 14.1)	60 (± 16.9)	0.063
PC aa C36:2	239 (± 40.5)	242.4 (± 47.9)	0.477
PC aa C36:3	130.1 (± 26.3)	131.6 (± 29.2)	0.695
PC aa C36:4	171.2 (± 37.6)	174 (± 39.2)	0.495
PC aa C36:5	33.3 (± 14.9)	31.6 (± 13.7)	0.505
PC aa C36:6	1.1 (± 0.4)	1.1 (± 0.5)	0.721
PC aa C38:0	2.5 (± 0.6)	2.5 (± 0.8)	0.761
PC aa C38:1	0.6 (± 0.3)	0.5 (± 0.4)	0.737
PC aa C38:3	49.6 (± 14.6)	52.4 (± 16.1)	0.06
PC aa C38:4	98.9 (± 28)	101.9 (± 30.7)	0.128
PC aa C38:5	54.5 (± 15.4)	54.8 (± 16)	0.84
PC aa C38:6	80.7 (± 23.6)	82.6 (± 25)	0.542
PC aa C40:2	0.3 (± 0.1)	0.2 (± 0.1)	0.032
PC aa C40:3	0.5 (± 0.1)	0.4 (± 0.1)	0.011
PC aa C40:4	2.6 (± 0.7)	2.8 (± 0.9)	0.077
PC aa C40:5	7.8 (± 2.5)	8.3 (± 2.7)	0.065
PC aa C40:6	24.1 (± 8.2)	25.3 (± 8.7)	0.183
PC aa C42:0	0.4 (± 0.1)	0.4 (± 0.1)	0.456
PC aa C42:1	0.2 (± 0)	0.2 (± 0.1)	0.974
PC aa C42:2	0.2 (± 0)	0.2 (± 0)	0.35
PC aa C42:4	0.1 (± 0)	0.1 (± 0)	0.419
PC aa C42:5	0.3 (± 0.1)	0.3 (± 0.1)	0.422
PC aa C42:6	0.5 (± 0.1)	0.4 (± 0.1)	0.63
PC ae C30:0	0.4 (± 0.2)	0.4 (± 0.2)	0.837
PC ae C30:1	0.2 (± 0.1)	0.3 (± 0.2)	0.345
PC ae C30:2	0.1 (± 0)	0.1 (± 0)	0.955
PC ae C32:1	3.1 (± 0.7)	3.1 (± 0.8)	0.975
PC ae C32:2	0.8 (± 0.2)	0.8 (± 0.2)	0.768
PC ae C34:0	1.6 (± 0.5)	1.6 (± 0.5)	0.827
PC ae C34:1	11.6 (± 2.7)	11.8 (± 2.8)	0.468
PC ae C34:2	13 (± 2.4)	13.1 (± 2.8)	0.74
PC ae C34:3	8.8 (± 2.4)	8.6 (± 2.5)	0.394
PC ae C36:0	0.8 (± 0.2)	0.9 (± 0.2)	0.517
PC ae C36:1	8.7 (± 2)	8.6 (± 1.9)	0.594
PC ae C36:2	15.3 (± 3.1)	14.8 (± 2.8)	0.209
PC ae C36:3	7.4 (± 1.4)	7.5 (± 1.5)	0.719
PC ae C36:4	16.1 (± 3.3)	16.5 (± 4.1)	0.488
PC ae C36:5	10.8 (± 2.2)	10.9 (± 3)	0.812
PC ae C38:0	2.3 (± 0.7)	2.2 (± 0.7)	0.202
PC ae C38:1	0.4 (± 0.2)	0.4 (± 0.2)	0.496
PC ae C38:2	1.8 (± 0.5)	1.8 (± 0.5)	0.622
PC ae C38:3	3.9 (± 1)	3.9 (± 0.8)	0.64
PC ae C38:4	12.1 (± 2.6)	12.2 (± 2.6)	0.81
PC ae C38:5	17 (± 3.2)	17.1 (± 3.5)	0.746
PC ae C38:6	7.3 (± 1.8)	7.4 (± 2.1)	0.818
PC ae C40:1	1.2 (± 0.2)	1.2 (± 0.3)	0.327

PC ae C40:2	2.1 (± 0.5)	2 (± 0.5)	0.32
PC ae C40:3	1 (± 0.2)	1 (± 0.2)	0.037
PC ae C40:4	1.9 (± 0.4)	1.9 (± 0.4)	0.338
PC ae C40:5	2.9 (± 0.6)	2.9 (± 0.6)	0.934
PC ae C40:6	4.2 (± 1.1)	4.2 (± 1.1)	0.789
PC ae C42:1	0.3 (± 0)	0.3 (± 0.1)	0.634
PC ae C42:2	0.5 (± 0.1)	0.5 (± 0.1)	0.245
PC ae C42:3	0.7 (± 0.1)	0.7 (± 0.1)	0.555
PC ae C42:4	0.6 (± 0.1)	0.6 (± 0.1)	0.911
PC ae C42:5	1.6 (± 0.3)	1.6 (± 0.4)	0.792
PC ae C44:3	0.1 (± 0)	0.1 (± 0)	0.637
PC ae C44:4	0.3 (± 0.1)	0.3 (± 0.1)	0.171
PC ae C44:5	1.2 (± 0.3)	1.2 (± 0.4)	0.378
PC ae C44:6	0.8 (± 0.2)	0.9 (± 0.2)	0.216
**Triacylglycerols**			
TG(46:2)	3.5 (± 2.9)	3.7 (± 3.8)	0.939
TG(48:0)	4.5 (± 4.6)	5.4 (± 5.1)	0.184
TG(48:1)	29.1 (± 26)	34.5 (± 29.1)	0.219
TG(48:2)	17.1 (± 11.9)	18.9 (± 14)	0.343
TG(48:3)	3.8 (± 2.6)	3.9 (± 2.7)	0.934
TG(49:1)	0.9 (± 0.8)	1 (± 1)	0.522
TG(49:2)	1 (± 0.8)	1.1 (± 1)	0.226
TG(50:1)	5.8 (± 3.8)	6.6 (± 4.3)	0.2
TG(50:2)	31.2 (± 16.9)	33.8 (± 19.6)	0.294
TG(50:3)	22.3 (± 12.6)	22.3 (± 13.2)	0.988
TG(50:4)	6.8 (± 4.3)	6.5 (± 4.2)	0.637
TG(52:4)	0.8 (± 0.4)	0.8 (± 0.4)	0.82
TG(52:5)	0.7 (± 0.4)	0.7 (± 0.4)	0.96
TG(44:1)	3.1 (± 4.5)	4.7 (± 6.1)	0.128
TG(44:2)	1.2 (± 1.4)	1.2 (± 1.3)	0.197
TG(46:2)	2.9 (± 2.8)	4.2 (± 5)	0.17
TG(48:0)	26 (± 33.1)	30.8 (± 29)	0.041
TG(48:1)	51.1 (± 48.1)	61 (± 52.8)	0.146
TG(48:2)	22.7 (± 18.2)	25.8 (± 21.7)	0.526
TG(48:3)	3.7 (± 2.9)	4 (± 3.4)	0.939
TG(49:1)	8.1 (± 6.9)	9 (± 7.8)	0.271
TG(49:2)	3 (± 2.1)	3.3 (± 2.5)	0.326
TG(50:0)	19.4 (± 17.2)	23.3 (± 17.4)	0.028
TG(50:1)	146.4 (± 105.7)	171.3 (± 111.7)	0.07
TG(50:2)	119.8 (± 67.4)	130.5 (± 76.9)	0.481
TG(50:3)	33.9 (± 17.6)	34.8 (± 19.6)	0.706
TG(50:4)	3.9 (± 2.3)	3.8 (± 2.4)	0.64
TG(51:1)	4.7 (± 3.6)	5.4 (± 4.2)	0.117
TG(51:2)	6.4 (± 3.9)	7.1 (± 4.5)	0.065
TG(51:3)	2.2 (± 1.2)	2.3 (± 1.3)	0.267
TG(52:2)	284 (± 123.2)	312.4 (± 146.4)	0.107
TG(52:3)	229.8 (± 100.9)	235.8 (± 120.3)	0.716
TG(52:4)	69.4 (± 37.8)	66.1 (± 41.9)	0.55
TG(52:5)	10.6 (± 6.2)	9.6 (± 5.7)	0.305
TG(52:6)	1.4 (± 0.9)	1.2 (± 0.6)	0.175
TG(53:3)	0.5 (± 0.3)	0.5 (± 0.3)	0.297
TG(54:1)	1.3 (± 0.8)	1.4 (± 0.9)	0.475

TG(54:2)	3.2 (± 1.7)	3.6 (± 1.8)	0.138
TG(54:3)	4.6 (± 2.4)	4.9 (± 2.9)	0.287
TG(54:4)	6.2 (± 3.6)	6.8 (± 3.7)	0.315
TG(54:5)	6.9 (± 4)	6.8 (± 3.7)	0.939
TG(54:6)	4.2 (± 2.8)	3.9 (± 1.9)	0.645
TG(54:7)	1.5 (± 1.1)	1.3 (± 0.7)	0.353
TG(56:6)	4.7 (± 2.8)	4.7 (± 2.6)	0.826
TG(56:7)	5 (± 4)	4.6 (± 2.2)	0.986
TG(56:8)	1.9 (± 1.6)	1.7 (± 0.9)	0.534
TG(44:1)	1.2 (± 1.2)	1.7 (± 2.4)	0.144
TG(46:2)	2.7 (± 2.8)	3.8 (± 4.2)	0.367
TG(48:1)	9.6 (± 8.4)	10.5 (± 9.3)	0.275
TG(48:2)	16.4 (± 12.7)	17.7 (± 14.7)	0.837
TG(48:3)	4.7 (± 2.9)	4.5 (± 3.3)	0.906
TG(49:2)	2.5 (± 1.8)	2.5 (± 1.8)	0.637
TG(50:1)	7.7 (± 5.3)	8.6 (± 6)	0.188
TG(50:2)	65.5 (± 38.9)	71.1 (± 45.2)	0.196
TG(50:3)	33.5 (± 17.5)	33.5 (± 19.2)	0.988
TG(50:4)	7.2 (± 4)	6.7 (± 3.7)	0.3
TG(52:2)	7.6 (± 4)	8 (± 4.6)	0.374
TG(52:3)	41.2 (± 19.9)	41.2 (± 22.3)	0.994
TG(52:4)	30.7 (± 15.6)	27.8 (± 15.8)	0.264
TG(52:5)	9.3 (± 5)	8.1 (± 4.8)	0.1
TG(52:6)	2 (± 1.2)	1.6 (± 0.9)	0.044
TG(54:4)	0.7 (± 0.3)	0.7 (± 0.4)	0.94
TG(54:5)	1.1 (± 0.6)	1 (± 0.6)	0.503
TG(54:6)	1.5 (± 0.7)	1.4 (± 0.6)	0.091
TG(49:1)	1.6 (± 1.3)	1.9 (± 1.6)	0.604
TG(51:1)	4.4 (± 3.3)	4.8 (± 3.3)	0.264
TG(51:2)	3.1 (± 1.9)	3.3 (± 2.1)	0.283
TG(51:3)	0.8 (± 0.3)	0.7 (± 0.3)	0.851
TG(53:3)	4.1 (± 1.8)	4 (± 1.9)	0.741
TG(53:4)	1.5 (± 0.7)	1.4 (± 0.7)	0.434
TG(49:2)	1.8 (± 1.1)	1.9 (± 1.4)	0.328
TG(51:2)	5.7 (± 3.6)	6.2 (± 3.9)	0.129
TG(51:3)	3 (± 1.6)	3 (± 1.8)	0.585
TG(51:4)	0.7 (± 0.3)	0.7 (± 0.3)	0.393
TG(53:4	2.3 (± 1)	2.2 (± 1)	0.607
TG(53:5)	0.8 (± 0.4)	0.7 (± 0.3)	0.266
TG(53:6)	0.4 (± 0.1)	0.3 (± 0.1)	0.168
TG(55:6)	0.1 (± 0.1)	0.1 (± 0.1)	0.489
TG(55:7)	0.1 (± 0.1)	0.1 (± 0.1)	0.907
TG(51:4)	0.3 (± 0.2)	0.3 (± 0.1)	0.767
TG(51:5)	0.3 (± 0.1)	0.3 (± 0.1)	0.464
TG(53:4)	0.4 (± 0.2)	0.5 (± 0.2)	0.322
TG(53:5)	0.4 (± 0.2)	0.4 (± 0.1)	0.551
TG(53:6)	0.5 (± 0.2)	0.5 (± 0.3)	0.563
TG(55:7)	0.2 (± 0.1)	0.2 (± 0.1)	0.436
TG(55:8)	0.2 (± 0.1)	0.2 (± 0.1)	0.079
TG(55:9)	0.1 (± 0)	0.1 (± 0.1)	0.558
TG(48:0)	3.1 (± 3.3)	3.7 (± 3.2)	0.24
TG(48:1)	1.5 (± 1.6)	2.0 (± 2.1)	0.121

TG(50:0)	4.5 (± 5.4)	5.7 (± 5.4)	0.046
TG(50:1)	5.8 (± 5.4)	7.1 (± 5.9)	0.059
TG(50:2)	1.8 (+ 1.3)	2.1 (± 1.5)	0.282
TG(52:2)	11.2 (± 6.8)	13.3 (± 8.7)	0.065
TG(52:3)	2.6 (± 1.3)	2.9 (± 1.6)	0.156
TG(54:1)	3.7 (± 2.5)	5.2 (± 6)	0.082
TG(54:2)	16.9 (± 8.3)	21.1 (± 14.9)	0.019
TG(54:3)	17.2 (± 7.8)	18.7 (± 10.7)	0.224
TG(54:4)	6.1 (± 3.2)	6.3 (± 3.7)	0.659
TG(54:5)	1.2 (± 0.5)	1.1 (± 0.5)	0.55
TG(54:6)	1.3 (± 0.6)	1.2 (± 0.6)	0.834
TG(54:7)	0.4 (± 0.2)	0.4 (± 0.2)	0.305
TG(44:1)	2.9 (± 3.7)	4.5 (± 6.5)	0.179
TG(44:2)	3.4 (± 3.3)	4.4 (± 4.7)	0.29
TG(48:1)	38.4 (± 33.8)	45.1 (± 37.3)	0.219
TG(48:2)	23.8 (± 17.3)	28.9 (± 23)	0.168
TG(48:3)	3.8 (± 2.3)	4.6 (± 3.4)	0.185
TG(49:1)	7.9 (± 6.4)	8.3 (± 7.1)	0.511
TG(50:2)	92.8 (± 64.5)	107.5 (± 69.8)	0.075
TG(50:2)	130.7 (± 69.2)	142.2 (± 79.4)	0.164
TG(50:3)	31.8 (± 16.2)	32.3 (± 17.9)	0.819
TG(50:4)	3.9 (± 2.2)	3.8 (± 2.1)	0.914
TG(51:1)	5.8 (± 4.1)	6.4 (± 4.3)	0.226
TG(51:2)	17.4 (± 9.2)	18.5 (± 9.7)	0.217
TG(51:3)	5.3 (± 2.8)	5.3 (± 2.7)	0.83
TG(51:4)	1.1 (± 0.6)	1 (± 0.6)	0.124
TG(52:2)	459.3 (± 184.1)	501.3 (± 213.9)	0.101
TG(52:3)	259.4 (± 106.9)	268.6 (± 127.4)	0.883
TG(52:4)	49 (± 23.5)	46.5 (± 24.2)	0.508
TG(52:5)	5.6 (± 3)	4.8 (± 2.4)	0.084
TG(53:3)	7.6 (± 3.6)	7.8 (± 3.7)	0.712
TG(53:4)	2.3 (± 1.2)	2.2 (± 1.1)	0.501
TG(54:1)	4.3 (± 2.1)	5.4 (± 3.8)	0.02
TG(54:2)	43.8 (± 19.9)	51.3 (± 30.2)	0.048
TG(54:3)	156.6 (± 77)	166.4 (± 89.5)	0.362
TG(54:4)	117.6 (± 64.9)	114.3 (± 68.6)	0.701
TG(54:5)	42.2 (± 27.1)	37.8 (± 24.9)	0.224
TG(54:6)	10.1 (± 7.1)	8.5 (± 6)	0.16
TG(54:7)	1.6 (± 1.1)	1.4 (± 0.7)	0.087
TG(56:6)	8.2 (± 3.8)	7.9 (± 3.3)	0.517
TG(56:7)	5.4 (± 3.8)	4.9 (± 2.0)	0.718
TG(56:8)	1.4 (± 1)	1.2 (± 0.5)	0.213
TG(46:2)	2.9 (± 2.6)	4.3 (± 4.8)	0.206
TG(48:2)	12.9 (± 9.7)	14.6 (± 11.2)	0.985
TG(48:3)	7.4 (± 4.8)	8.4 (± 6.2)	0.318
TG(50:2)	35.7 (± 20.4)	39.4 (± 23.6)	0.5
TG(50:3)	45.8 (± 23.5)	45.6 (± 23.8)	0.969
TG(50:4)	11.4 (± 6.7)	10.4 (± 6.3)	0.399
TG(51:2)	2.2 (± 1.3)	2.3 (± 1.4)	0.413
TG(51:3)	6.2 (± 3.6)	6.1 (± 3.5)	0.719
TG(51:4)	2.1 (± 1.3)	1.9 (± 0.9)	0.097
TG(52:2)	24.9 (± 12)	27 (± 14.7)	0.221

TG(52:3)	205.3 (± 93.8)	215.4 (± 113)	0.494
TG(52:4)	106.7 (± 59.5)	100.8 (± 67.2)	0.548
TG(52:5)	18.2 (± 10.6)	15.7 (± 10.5)	0.147
TG(52:6)	2.1 (± 1.3)	1.7 (± 1)	0.035
TG(53:3)	4.3 (± 2.2)	4.3 (± 2.1)	0.791
TG(53:4)	2.6 (± 1.4)	2.5 (± 1.2)	0.363
TG(53:5)	0.9 (± 0.5)	0.7 (± 0.4)	0.113
TG(54:2)	2.4 (± 1)	2.8 (± 1.7)	0.176
TG(54:3)	21.5 (± 10.1)	23.2 (± 13)	0.241
TG(54:4)	59.6 (± 33.3)	58.5 (± 36)	0.808
TG(54:5)	39.8 (± 29.5)	34.9 (± 26.8)	0.18
TG(54:6)	16.7 (± 14.3)	13.4 (± 11.8)	0.073
TG(54:7)	4.1 (± 3.5)	3.2 (± 3.2)	0.071
TG(56:6)	2.4 (± 1.3)	2.2 (± 1.3)	0.247
TG(56:7)	3.6 (± 2)	3.2 (± 1.7)	0.063
TG(56:8)	2.5 (± 2)	2 (± 1)	0.066
TG(48:2)	2 (± 1.5)	2.3 (± 1.8)	0.488
TG(48:3)	4.8 (± 2.8)	5.1 (± 3.2)	0.507
TG(50:4)	7.1 (± 4.1)	6.7 (± 3.9)	0.594
TG(51:5)	0.3 (± 0.3)	0.3 (± 0.2)	0.048
TG(52:3)	3.5 (± 1.8)	3.5 (± 2)	0.947
TG(52:4)	28.8 (± 13.9)	28.5 (± 15.1)	0.881
TG(52:5)	15.7 (± 8.7)	13.9 (± 8.3)	0.228
TG(52:6)	2.6 (± 1.6)	2.2 (± 1.3)	0.136
TG(53:5)	0.6 (± 0.3)	0.5 (± 0.3)	0.127
TG(54:4)	3.4 (± 1.7)	3.2 (± 1.8)	0.576
TG(54:5)	13.2 (± 8.6)	12 (± 7.5)	0.349
TG(54:6)	8.7 (± 6.7)	7.4 (± 6.2)	0.25
TG(54:7)	3.2 (± 2.7)	2.6 (± 2.5)	0.168
TG(54:8)	0.6 (± 0.4)	0.5 (± 0.3)	0.061
TG(54:9)	0.5 (± 0.3)	0.4 (± 0.2)	0.018
TG(52:3)	0.6 (± 0.2)	0.7 (± 0.2)	0.075
TG(52:4)	0.6 (± 0.3)	0.5 (± 0.3)	0.57
TG(54:1)	1.3 (± 0.6)	1.7 (± 0.9)	0.172
TG(50:2)	0.3 (± 0.1)	0.3 (± 0.2)	0.624
TG(52:2)	1.1 (± 0.7)	1.1 (± 0.8)	0.618
TG(52:3)	0.5 (± 0.2)	0.5 (± 0.2)	0.876
TG(52:4)	0.3 (± 0.1)	0.2 (± 0.1)	0.093
TG(54:1)	0.6 (± 0.3)	0.6 (± 0.3)	0.616
TG(54:2)	3.2 (± 1.9)	3.4 (± 1.7)	0.313
TG(54:3)	1.8 (± 1)	1.8 (± 0.9)	0.971
TG(54:4)	0.4 (± 0.2)	0.4 (± 0.2)	0.295
TG(52:2)	1 (± 0.7)	1.1 (± 0.7)	0.2
TG(52:3)	1.2 (± 0.6)	1.3 (± 0.6)	0.146
TG(54:3)	3.2 (± 1.6)	3.4 (± 1.9)	0.179
TG(54:4)	2 (± 1)	2 (± 1.1)	0.784
TG(54:5)	0.4 (± 0.2)	0.4 (± 0.2)	0.68
TG(56:7)	0.1 (± 0.1)	0.1 (± 0)	0.907
TG(52:3)	1.5 (± 1.1)	1.8 (± 1.2)	0.111
TG(52:4)	2 (± 1.2)	2.2 (± 1.3)	0.225
TG(52:5)	0.6 (± 0.3)	0.6 (± 0.3)	0.747
TG(54:3)	0.9 (± 0.4)	0.9 (± 0.5)	0.017

TG(54:4)	6.9 (± 3.9)	7.4 (± 4.2)	0.222
TG(54:5)	4 (± 2.3)	3.9 (± 2.3)	0.771
TG(54:6)	0.8 (± 0.5)	0.8 (± 0.4)	0.386
TG(56:6)	2 (± 1)	1.9 (± 1.2)	0.504
TG(56:7)	0.8 (± 0.4)	0.7 (± 0.4)	0.23
TG(56:8)	0.4 (± 0.1)	0.3 (± 0.1)	0.239
TG(50:4)	1.2 (± 1)	1.5 (± 1.5)	0.488
TG(52:4)	2.7 (± 1.7)	3.1 (± 2.2)	0.148
TG(52:5)	3.2 (± 1.9)	3.5 (± 2.5)	0.325
TG(52:6)	1.1 (± 0.7)	1.1 (± 0.7)	0.676
TG(53:6)	0.2 (± 0.1)	0.2 (± 0.1)	0.879
TG(54:4)	1.9 (± 1)	2.1 (± 1.1)	0.053
TG(54:5)	14.4 (± 7.7)	15.1 (± 7.5)	0.306
TG(54:6)	8.4 (± 4.8)	8 (± 4.2)	0.442
TG(54:7)	1.6 (± 1)	1.3 (± 0.7)	0.025
TG(55:7)	0.1 (± 0)	0.1 (± 0)	0.242
TG(56:6)	9.1 (± 4.4)	8.7 (± 4)	0.268
TG(56:7)	5.3 (± 2.7)	4.8 (± 2.4)	0.057
TG(56:8)	2 (± 1.1)	1.7 (± 0.8)	0.009
TG(56:9)	0.6 (± 0.3)	0.6 (± 0.3)	0.137
TG(54:5)	0.8 (± 0.6)	0.8 (± 0.4)	0.953
TG(54:6)	5.4 (± 4.6)	4.5 (± 2.2)	0.327
TG(54:7)	3.3 (± 3)	2.5 (± 1.2)	0.018
TG(56:7)	3 (± 2.4)	2.4 (± 1.1)	0.099
TG(56:8)	2.1 (± 1.8)	1.5 (± 0.7)	0.004
TG(54:6)	0.01 (± 0)	0.01 (± 0)	0.649
TG(54:6)	0.1 (± 0.1)	0.1 (± 0.1)	0.173
TG(54:4)	0.4 (± 0.3)	0.4 (± 0.3)	0.155
TG(54:6)	0.1 (± 0.1)	0.1 (± 0)	0.268
TG(56:6)	1.1 (± 0.7)	1.1 (± 0.6)	0.603
TG(54:5)	1.3 (± 0.8)	1.3 (± 0.8)	0.573
TG(54:6)	1.4 (± 0.7)	1.4 (± 0.7)	0.974
TG(56:6)	7.1 (± 3.8)	7.1 (± 3.3)	0.994
TG(56:7)	4.1 (± 2.3)	3.7 (± 1.8)	0.217
TG(56:8)	0.9 (± 0.5)	0.7 (± 0.4)	0.058
TG(54:6)	3 (± 2.7)	2.9 (± 1.9)	0.91
TG(54:7)	3.6 (± 2.7)	3.4 (± 2.2)	0.836
TG(56:7)	15.8 (± 15.5)	14.1 (± 8.4)	0.727
TG(56:8)	9.1 (± 9.2)	7.4 (± 4.3)	0.754
TG(56:9)	1.8 (± 1.7)	1.4 (± 0.8)	0.842
**Various Metabolites**			
Beta-Alanine	2.5 (± 0.7)	2.4 (± 0.6)	0.047
AABA	17.4 (± 6.8)	16.4 (± 4.7)	0.162
HArg	3 (± 1.3)	2.8 (± 1.4)	0.08
HCys	6.2 (± 1.6)	6.4 (± 1.8)	0.133
SDMA	0.7 (± 0.2)	0.6 (± 0.2)	0.136
AA	4.3 (± 1.4)	3.6 (± 0.9)	0.007
DHA	6.2 (± 2.9)	5.4 (± 2.7)	0.1
EPA	0.9 (± 0.4)	0.7 (± 0.3)	0.006
3-IAA	1.9 (± 0.8)	1.9 (± 0.6)	0.452
3-IPA	4.9 (± 18.3)	3.1 (± 10.9)	0.129
Ind-SO4	5.7 (± 3.2)	6.1 (± 2.5)	0.188

H1	4512.9 (± 465)	4475.3 (± 475.7)	0.377
TMAO	7.1 (± 11.5)	8.7 (± 17.4)	0.967

Data are presented as mean (µmol/l) ± standard error of mean (SEM).

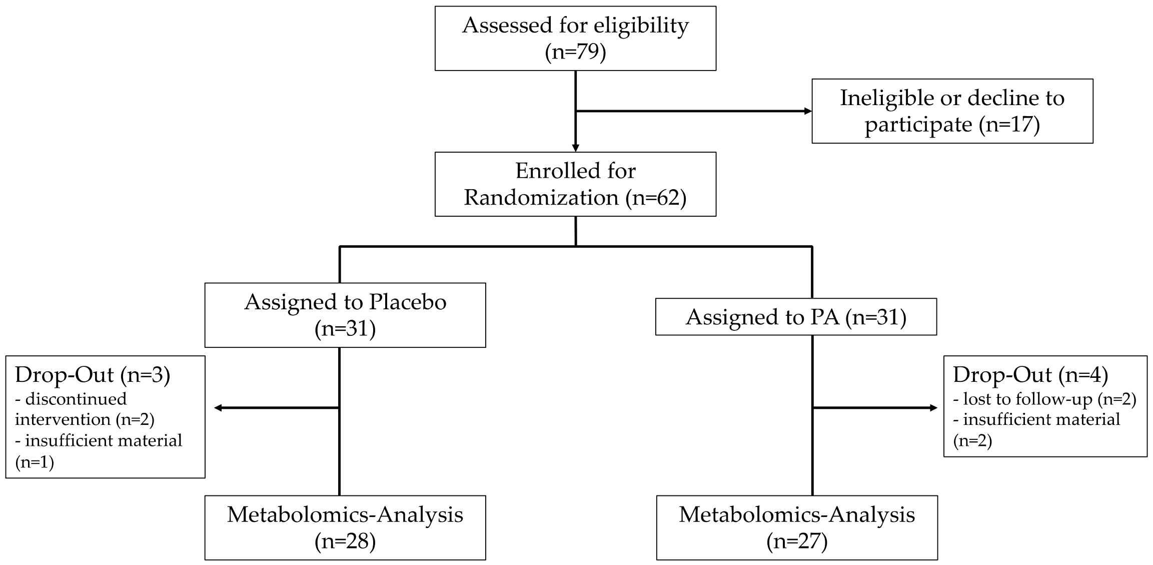

Supplementary Figure S1. Study design.A total of 62 patients were initially enrolled for 1:1 randomization and 58 patients completed the original study. Sufficient serum material from 55 patients (placebo: n = 28, PA = 27) was obtained for further metabolomic analysis in this study.
